# Bioinspired quantum dots: advancing diagnostic and therapeutic strategies in breast cancer

**DOI:** 10.1039/d5ra03443d

**Published:** 2025-08-04

**Authors:** Soji Soman, Sanjay Kulkarni, Farhath Sherin, Amrita Arup Roy, Anoushka Mukharya, Rahul Pokale, Srinivas Mutalik

**Affiliations:** a Department of Pharmaceutics, Manipal College of Pharmaceutical Sciences, Manipal Academy of Higher Education Manipal 576104 Karnataka India ss.mutalik@manipal.edu; b Department of Pharmaceutics, SRM College of Pharmacy, SRM Institute of Science and Technology Kattankulathur 603203 Chengalpattu Tamil Nadu India

## Abstract

Bioinspired quantum dots (BQDs) have garnered significant attention in recent years because of their unique characteristics, including their nanoscale size (less than 10 nm), high surface area, photoluminescence, chemical stability, and ease of synthesis and functionalization. Researchers are increasingly shifting towards the use of biomass-derived precursors instead of chemical compounds for BQD fabrication. These biomass sources are sustainable, eco-friendly, cost effective, widely available, and enable the conversion of waste into valuable materials. In this review, we provide a comprehensive analysis of various fabrication methodologies for BQDs, and the diverse raw materials used in recent studies. Owing to their exceptional properties, combined with simple synthesis routes, BQDs are promising candidates for a range of biomedical applications, particularly in bioimaging, targeted drug delivery, and phototherapy for cancer treatment. BQDs exhibit excellent aqueous solubility, low toxicity, biocompatibility, facile biofunctionalization, and selective cancer targeting. Furthermore, their photoluminescent properties, high longitudinal relaxation values, photothermal effects upon laser irradiation, ability to generate singlet oxygen, and production of H_2_S for gas therapy make them highly effective as cancer theranostic agents. This review specifically highlights the potential of BQDs in breast cancer management while addressing existing challenges in their application.

## Abstract 2

Bioinspired quantum dots offer a green approach for breast cancer theranostics, enabling targeted imaging, drug delivery, and phototherapy.

## Graphical abstract

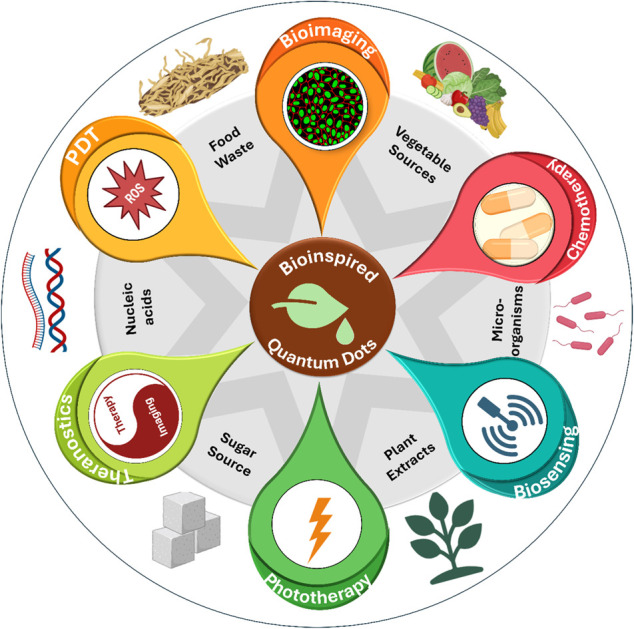

## 1. Introduction

Breast cancer (BC) is a common type of malignant cancer that affects millions of women worldwide. According to a study by the American Cancer Society, an estimated 1.9 million new cancer cases are predicted to be diagnosed in the United States in 2022, along with 609 360 cancer-related deaths.^1^ Conventional treatment regimens for BC encompass various approaches, including chemo-, immuno-, radio- and hormonal therapies. However, each of these methods has limitations, such as insufficient target-mediated targeting, improper biodistribution of therapeutics, limited efficiency, stability problems in biological environments, and associated toxic side effects.^2^

The heterogeneity of breast cancer, along with the intricate pathophysiological changes that occur during metastasis, underscores the necessity for precision medicine in effective treatment. There is also an important necessity to develop efficient drug delivery systems that can enhance the therapeutic potential of anticancer medications while minimizing their drawbacks. To address these challenges, nanoplatforms and nanomedicines have emerged as promising therapeutic options for breast tumor management. These advanced systems provide solutions to the limitations of conventional drugs and facilitate targeted chemotherapeutic delivery to various regions of breast cancer, such as the tumor vasculature, stromal cells, tumor cells, and immune cells.^3,4^

Conventional cancer therapies face several significant limitations. One major drawback is their lack of specificity in targeting cancer cells, which often results in damage to normal, rapidly proliferating cells and fails to achieve the desired therapeutic outcomes in many cases. The lack of selective targeting for cancer cells can result in significant side effects, including damage to healthy organs. Moreover, certain chemotherapeutic agents are rapidly cleared from circulation due to macrophage-mediated engulfment, which shortens their circulation time and limits their interaction with cancer cells, ultimately reducing their therapeutic effectiveness. Another hurdle is multidrug resistance, which significantly undermines the efficacy of conventional treatments. Furthermore, many chemotherapeutic agents are hydrophobic, resulting in poor aqueous solubility and low bioavailability, which further restricts their therapeutic potential.^5,6^

The growing demands of humanity have driven the development of innovative approaches that enhance convenience while safeguarding the environment from degradation. Among these advancements, nanotechnology stands out as a leading field, leveraging materials with dimensions in the nanoscale range of 1–100 nm. These exceptionally small nanoplatforms possess an improved surface-to-volume ratio, enabling them to display unique characteristics unattainable by bulk materials. While numerous types of nanomaterials are available, quantum dots (QDs) have recently attracted substantial attention, especially following the rise of metal-based nanoparticles. QDs, also referred to as semiconductor nanomaterials, are distinguished by their unique optical and electrical characteristics, which depend on their particle size. In addition to their applications in diverse fields, such as robotics, aeronautics, environmental science, and agriculture, QDs exhibit remarkable potential in the biomedical domain. They are particularly effective in diagnosing a variety of diseases, including neurodegenerative disorders and cancer. Quantum dots are crystalline, semiconducting nanoparticles or luminous nanocrystals (1–10 nm), and their characteristics are intrinsically linked to their size.^7^

Biogenically synthesized, bioinspired QDs (BQDs) are a promising category of fluorescent bionanomaterials produced from natural biomaterials. Compared with traditional QDs, they have attracted considerable interest because of their eco-friendly behavior, biocompatibility, improved water solubility, luminescence, and versatile functionality. Compared with standard drugs, BQDs strongly interact with phosphate moieties on DNA and display superior tumor inhibition, along with selective cytotoxicity toward cancer cells.^8–10^ Owing to their size-dependent photoluminescent properties, biogenically synthesized QDs hold considerable promise as tumor imaging agents.^11,12^ Furthermore, the combination of multiple imaging modalities has garnered attention for its ability to enhance tumor diagnosis while minimizing physiological stress.^13^ For example, doping BQDs with gadolinium (Gd) improves their longitudinal relaxivity, facilitating dual contrast imaging through both magnetic resonance and fluorescence techniques.^14^ This innovation allows for easier tracking of their *in vivo* behaviour than traditional nanocarriers do. Notably, BQDs have been employed as therapeutics within cells. Their optical absorbance in the near-infrared (NIR) region enables efficient photothermal energy conversion, which can be utilized to ablate tumors following their cellular uptake.^15,16^ However, despite their promising attributes, the ability of BQDs to affect cancer prognosis remains relatively underexplored.

**Fig. 1:**
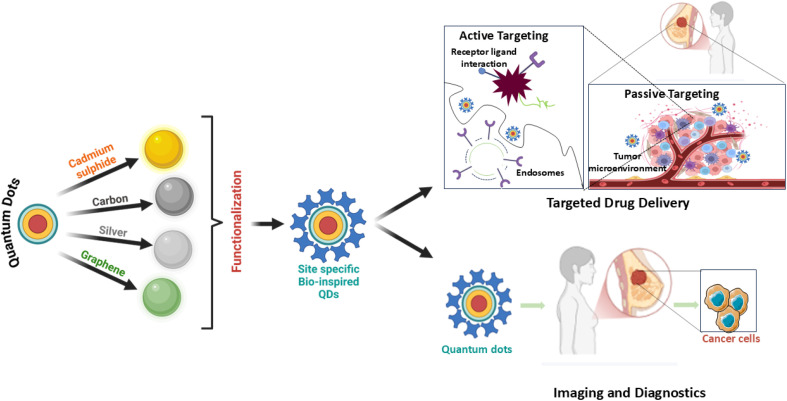
Schematic illustration representing synthesis and biomedical uses of bioinspired quantum dots.

Numerous studies have explored the biomedical applications of QDs across various domains, highlighting their application in imaging, chemotherapeutic delivery, and diagnostics.^17,18^ However, this article focuses on the innovative integration of plant-derived compounds with QDs for breast cancer therapy (Fig. 1). By combining the exceptional optical and electrical characteristics of QDs with the therapeutic benefits of bioactive plant compounds, this approach aims to develop more targeted and biocompatible treatments. This review uniquely provides a focused and comprehensive evaluation of BQDs in breast cancer management, emphasizing their ecofriendly synthesis from plants, microbes, and biomass waste and examining how surface functionalization impacts targeted delivery, phototherapy, immune modulation, and nanotheranostics. It critically assesses biosafety, clinical challenges, and translational barriers and outlines prospects such as machine learning and sustainable synthesis. By bridging green nanotechnology with precision breast cancer therapy, this review addresses a key gap in the current literature. This exploration seeks to inspire researchers and healthcare professionals to further investigate and harness this promising hybrid technology.

## 2. Properties of bio-inspired quantum dots

BQDs stand out as prominent nanomaterials for the treatment of breast cancer because of their exceptional physicochemical and biological attributes. In contrast to conventional QDs, which contain heavy metals such as cadmium, BQDs derived from natural plant extracts, biomolecules or carbon-based materials offer a greater biocompatible alternative with minimal toxicity risk.^19^ The exceptional size-dependent fluorescence property of BQDs makes them more effective and precise as imaging agents in cancer diagnosis. Additionally, the photoluminescence properties support real-time tumor tracking, assisting in precise drug delivery and effective monitoring of the therapeutic response. Moreover, their superior photothermal conversion efficiency allows them to absorb NIR light and generate localized heat, making them well-suited candidates for photothermal therapy (PTT) to eradicate cancer cells selectively.^20^ Another key advantage of BQDs is their remarkable functionalization capabilities with biomolecules such as targeting ligands, antibodies or small-molecule drugs to improve their specificity towards breast cancer cells. For example, breast cancer cells with more folate receptors efficiently take up folic acid-coated BQDs. Their surface chemistry can be engineered for pH-sensitive drug release, ensuring effective targeted drug release with minimal systemic toxicity.^20^

With the combination of both therapy and diagnostics properties of BQDs makes them highly effective in cancer treatment. It has been reported that these nanomaterials can deliver chemotherapeutic drug like doxorubicin as well as enable the fluorescence imaging to track drug distribution. This combination enhances the effectiveness of treatment at the same time enables the non-intrusive monitoring of therapeutic progress.^20^ With the exceptional eco-friendly synthesis methods, remarkable stability of carbon-based QDs offers excellent advantages in biomedical applications.^19^

BQDs exhibit unique optical properties, including strong UV absorption (210–360 nm), excitation-dependent emission, pH sensitivity, and upconversion photoluminescence. Their typical emission ranges from 400–500 nm and is influenced by the particle size, surface functional groups, and excitation wavelength. The presence of π–π* and n–π* transitions (*e.g.*, C=C, C=O) contributes to their optical activity. However, owing to the surface defects and heterogeneity inherent in biomass precursors, their photoluminescence efficiency is generally lower and less uniform than that of chemically synthesized QDs, which benefits from precise structural control.^21^ The choice of biomass precursor significantly influences the quantum yield (QY) and emission wavelength of BQDs because of differences in chemical composition, such as the contents of lignin, cellulose, and heteroatoms. For example, lignin-rich biomass, which contains more aromatic rings and heteroatoms, tends to produce BQDs with higher degrees of graphitization and can achieve higher quantum yields than cellulose-rich precursors. Studies have shown that CQDs derived from cellulose and lignin at different temperatures exhibit quantum yields of 11.7% and 23.4%, respectively, highlighting the impact of the precursor composition and structure.^21,22^

The nanoscale dimensions (<10 nm) of BQDs significantly influence their electronic band structure due to the quantum confinement effect. When materials are reduced to the nanometer scale in one or more dimensions, they begin to display remarkable and unique properties. A key phenomenon observed at this scale is the restriction of electron movement, known as the quantum confinement effect. This effect causes the energy levels of electrons to become discrete, with their spacing determined by the size of the confined region. As a result, nanostructured materials exhibit distinctive optoelectronic, physicochemical, mechanical, and magnetic behaviors that are absent in their bulk counterparts.^23^ Compared with their bulk counterparts, BQDs exhibit altered electron and hole wavefunction overlap, modified charge carrier dynamics, and enhanced surface-to-volume ratios, all of which are crucial for their high photostability, brightness, and tunability in biomedical imaging and theranostics. These features are particularly beneficial in breast cancer diagnostics, where precision and selectivity are vital.^24,25^

Different synthesis routes significantly influence the thermal and chemical stability of BQDs by affecting their crystallinity, surface chemistry, and structural uniformity. Bottom-up approaches such as hydrothermal, solvothermal, and solid-state methods typically yield BQDs with higher crystallinity, enhanced photostability, salt tolerance, and pH stability. These methods also allow better control over surface functionalization, improving dispersion and resistance to degradation in biological and aqueous environments.^26,27^ The chemical composition of biomass sources significantly influences the optical and stability characteristics of BQDs. As shown in the present study, variations in carbon, nitrogen, and oxygen contents from sources such as sugarcane bagasse, garlic peel, and taro peel resulted in QDs with different particle sizes and quantum yields. For example, taro peels with higher nitrogen contents produced QDs with the highest quantum yield (26.2%) and smallest particle size (0–2 nm), whereas sugarcane bagasse yielded larger particles with lower fluorescence (4.45%). These differences stem from the abundance of heteroatoms (*e.g.*, N, O) and functional groups that impact surface passivation, the photoluminescence intensity, and the stability of the dots during synthesis.^28^

## 3. Synthesis approaches

Biosynthesis approaches offer a greener and safer alternative to chemical methods, operating under mild conditions without the need for additional capping agents or encapsulation. Biomass-derived systems contain inherent metal-reducing and metal-binding biomolecules such as proteins, enzymes, and amino acids that facilitate controlled nucleation, size regulation, and surface passivation of QDs. These biomolecules help produce BQDs with intrinsic biocompatibility, stability, and tunable optical properties. The process is eco-friendly, cost-effective, scalable, and avoids toxic solvents and harsh reaction environments, making it suitable for biomedical applications.^29^

The top-down approach involves breaking down bulk carbon materials (*e.g.*, graphite and carbon nanotubes) *via* physical or chemical methods such as laser ablation or electrochemical oxidation. It offers good control over crystallinity and particle size but often requires harsh conditions and high energy input, resulting in lower yields and limited surface functionalization. In contrast, the bottom-up approach widely used for biomass-derived BQDs builds quantum dots from molecular precursors *via* hydrothermal, pyrolysis, or microwave methods. This method is simple, low-cost, and eco-friendly and enables better control over surface chemistry, making it ideal for scalable biomedical applications.^30^ The impurities present in biomass precursors, such as residual minerals, heavy metals, proteins, or polysaccharides, can significantly affect the emission wavelength, photoluminescence intensity, and quantum yield. Strict control over precursor purity and process consistency is essential to ensure reproducible, safe, and high-performance BQDs for biomedical applications.^16,21,29^ Furthermore, the composition of the biomass directly affects the yield and functionalization of the resulting BQDs. Biomass with a high content of usable carbon precursors typically allows for higher yields, whereas complex or variable compositions can lead to unpredictable or lower outputs.^31,32^

### 3.1. Synthesis using microorganisms

The biological synthesis of QDs using microbes has emerged as a green, cost-effective, and sustainable approach for nanomaterial production. Unlike traditional chemical methods, which often involve toxic precursors and high-energy inputs, microbial biosynthesis leverages intracellular and extracellular metabolic pathways to facilitate QD formation under ambient conditions. Bacteria, fungi, and yeast have demonstrated remarkable capabilities in producing cadmium sulfide (CdS), cadmium telluride (CdTe), zinc sulfide (ZnS), and cadmium selenide (CdSe) quantum dots, among others. These microbes employ various mechanisms, including enzymatic reduction, biomineralization, and metabolic transformation, to convert metal ions into stable, fluorescent nanocrystals with controlled size and morphology.

Among bacterial systems, *Pseudomonas putida* KT2440 was utilized to synthesize cadmium sulfide (CdS) quantum dots alongside medium-chain-length polyhydroxyalkanoates (MCL-PHAs). The bacterium was cultivated in M9 minimal medium for 48 to 68 hours before cadmium chloride (CdCl_2_) was introduced. l-cysteine was then added, promoting the biotransformation of toxic Cd^2+^ ions into CdS QDs *via* hydrogen sulfide (H_2_S) production. The QDs exhibited fluorescence under UV light, confirming their successful formation. Notably, CdS QDs are localized in the periplasmic space, whereas MCL-PHAs accumulate in the cytoplasm, indicating distinct biochemical compartmentalization.^33^ Similarly, *Escherichia coli* synthesized CdS QDs when cultivated in Luria–Bertani (LB) medium at 37 °C. After an initial growth phase of seven hours, the culture medium was replaced, and Cd^2+^ ions were introduced at 1.0 × 10^−3^ mol L^−1^. The bacterial metabolic pathways facilitated CdS QD formation over two days. Transmission electron microscopy (TEM) confirmed the presence of uniform particles (∼10 nm), and fluorescence analysis revealed peak emission at 470 nm. Notably, the antibiotic resistance profile of *E. coli* is maintained postsynthesis.^34^*Saccharomyces cerevisiae* MTCC 2918 facilitated the eco-friendly biosynthesis of ZnS QDs intracellularly when grown in YEPD broth. Upon exposure to 1 mM zinc sulfate (ZnSO_4_), yeast metabolic pathways facilitated Zn^2+^ ion reduction, resulting in the production of ZnS nanoparticles within 24 hours. The nanoparticles were extracted *via* freeze–thaw cycles and vortexing, which lysed the yeast cells to release the QDs into the solution.^35^

*Escherichia coli* K12 synthesizes CdTe QDs extracellularly through secreted proteins. The bacteria were cultured in LB medium until they reached an absorbance of 0.6 at 600 nm and then exposed to cadmium chloride (CdCl_2_), sodium tellurite (Na_2_TeO_3_), trisodium citrate, mercaptosuccinic acid (MSA), and sodium borohydride (NaBH_4_). After incubation, the supernatant containing the CdTe QDs was confirmed *via* fluorescence characterization.^36^ Yeast and fungi have also demonstrated significant potential for QD biosynthesis. *Phanerochaete chrysosporium*, a white rot fungus, produces CdS QDs when cultivated in Kirk inorganic liquid media. After mycelium pellets formed, cadmium nitrate tetrahydrate, thioacetamide (TAA), and mercaptoacetic acid were introduced. At pH 9.0–11.0 and 37 °C, biosynthesis occurred, as indicated by a visible color change in the mycelial pellets from white to yellow after 12 hours.^37^*Fusarium oxysporum* facilitated CdTe QD biosynthesis through a two-step process. First, the fungus was grown in MGYP media for 96 hours. The mycelia were then separated and incubated in a solution containing CdCl_2_ and TeCl_4_ (1 mM each) at 25–27 °C for another 96 hours. This method yielded CdTe QDs through fungal metabolism, demonstrating an environmentally friendly synthesis approach.^38^ The Antarctic bacterium *Pseudomonas fragi* GC01 utilizes volatile sulfur compounds (VSCs), such as H_2_S, methanethiol (MeSH), and dimethyl sulfide (DMS), for QD biosynthesis. When cultured with sulfate, cysteine, or methionine, the bacterium produced intracellular and extracellular CdS QDs. Mutant analysis of Pseudomonas deceptionensis M1T confirmed that MeSH was essential for CdS QD formation, whereas DMS was not.^39^

*Fusarium oxysporum* also enables the biosynthesis of CdSe QDs. After cultivation under optimized conditions, the fungus was exposed to cadmium chloride (CdCl_2_) and selenium ions (Na_2_SeO_3_ or Na_2_SeO_4_) at 5 mM concentrations and pH 7.5. At 25 °C, intracellular CdSe QDs formed within the fungal cells. Proteomic analysis revealed increased superoxide accumulation, indicating a role in selenium ion reduction and QD synthesis.^40^ To further extend their biosynthesis capabilities, *E. coli* synthesized ternary CdSAg QDs by initially producing CdS QDs in the presence of cysteine and CdCl_2_. Silver nitrate (AgNO_3_) was subsequently introduced (15–200 μM), facilitating a cation exchange process wherein Cd^2+^ was partially replaced by Ag^+^, yielding near-infrared fluorescent CdSAg QDs.^41^ Finally, *Halobacillus* sp. DS2, a polyextremophile, synthesized CdS QDs under hypersaline conditions (3–22% NaCl). When cultured with cadmium chloride and cysteine, the bacterium produced H_2_S *via* cysteine desulfhydrases, which reacted with Cd^2+^ to form extracellular and intracellular CdS nanoparticles.^42^

### 3.2. Synthesis using plant extracts

**Fig. 2:**
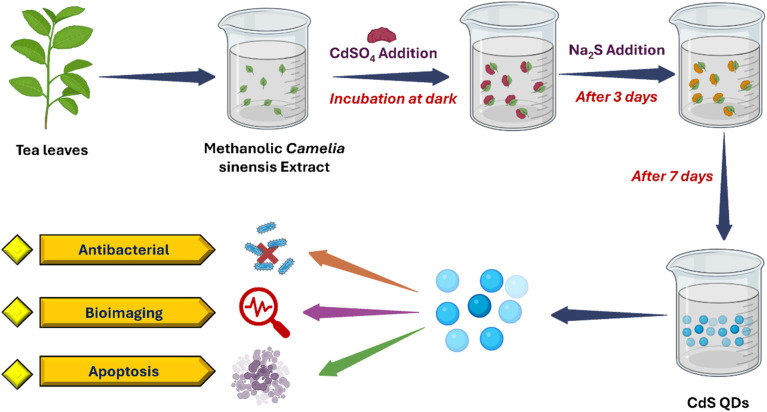
Diagrammatic representation of the experimental stages involved in the green synthesis of CdS quantum dots (QDs) using *Camellia sinensis* extract and its biomedical applications.

Plant-based biosynthesis leverages the natural reducing, stabilizing, and capping agents found in plant phytochemicals such as polyphenols, flavonoids, alkaloids, and terpenoids. These bioactive compounds facilitate metal ion reduction and nanoparticle stabilization, allowing the controlled formation of quantum dots with tailored sizes, crystallinities, and fluorescence properties. Several studies have successfully demonstrated the green synthesis of cadmium sulfide (CdS), silver (Ag), zinc oxide (ZnO), and carbon quantum dots (CQDs) from diverse plant extracts. The biosynthesis of cadmium sulfide (CdS) QDs *via* the use of waste-matured tea leaves as biosurfactants represents a significant advancement in green nanotechnology. The process began with the collection of discarded mature tea leaves, which were washed to remove impurities, chopped, and mixed with methanol at a 1 : 10 ratio. After filtration, a clear tea leaf extract rich in phytochemicals was obtained. This extract was then added to a mixture of cadmium sulfate (CdSO_4_) and sodium sulfide (Na_2_S), stirred, and incubated in the dark for one week. The incubation period facilitated the formation of CdS QDs with a cubic crystalline structure, measuring between 2.5 and 4 nm. A color change in the solution confirmed the successful synthesis of the CdS QDs.^43^ A similar biogenic approach was employed for synthesizing CdS QDs using Camellia sinensis extract as a stabilizing agent, as illustrated in Fig. 2. The tea leaves were collected, washed, shade-dried, and processed into an extract using methanol. The biosynthesis occurred in two stages: cadmium sulfate was initially incubated with the extract in the dark for three days, followed by the addition of sodium sulfide. This reaction continued for four more days, leading to the formation of bright yellow CdS QDs. The particles were then purified through centrifugation and lyophilization for characterization.^44^

To expand green synthesis methods, silver quantum dots (Ag QDs) were synthesized from Citrus limetta (sweet lime) peel extract. The peels were boiled in water to extract bioactive compounds, and the extract was mixed with silver nitrate (AgNO_3_) solution. The mixture was then refluxed at various temperatures, facilitating the reduction of silver ions into Ag QDs. A surface plasmon resonance (SPR) peak at 415 nm confirmed successful synthesis.^45^ Similarly, ZnO QDs were synthesized *via* a microwave-assisted technique from *Catharanthus roseus* leaf extract and Aloe vera gel. The plant extracts were mixed with zinc sulfate and subjected to microwave radiation for 15 minutes. The fluorescence properties of the ZnO QDs were verified under UV light, demonstrating an eco-friendly synthesis method.^46^

Another CdS QD biosynthesis approach utilized extracts from green cardamom and ginger. The plant materials were washed, air-dried, and processed into fine powder and grated forms. A mixture of methanol and deionized water was used for extraction, followed by incubation with cadmium sulfate and sodium sulfide. The reaction proceeded for six hours and was stored in the dark for four days to ensure complete QD formation. After purification and drying, CdS QDs with improved biocompatibility were obtained.^47^ In another case, CdS QDs were synthesized *via* hairy root extracts from *Rhaphanus sativus*. The roots were cultivated in hormone-free medium *via* Agrobacterium rhizogenes and processed into an aqueous extract. This extract was mixed with cadmium sulfate and incubated in the dark, followed by the addition of sodium sulfide. The reaction yielded yellowish CdS QDs, which were purified through centrifugation, demonstrating an environmentally friendly method.^48^

**Fig. 3:**
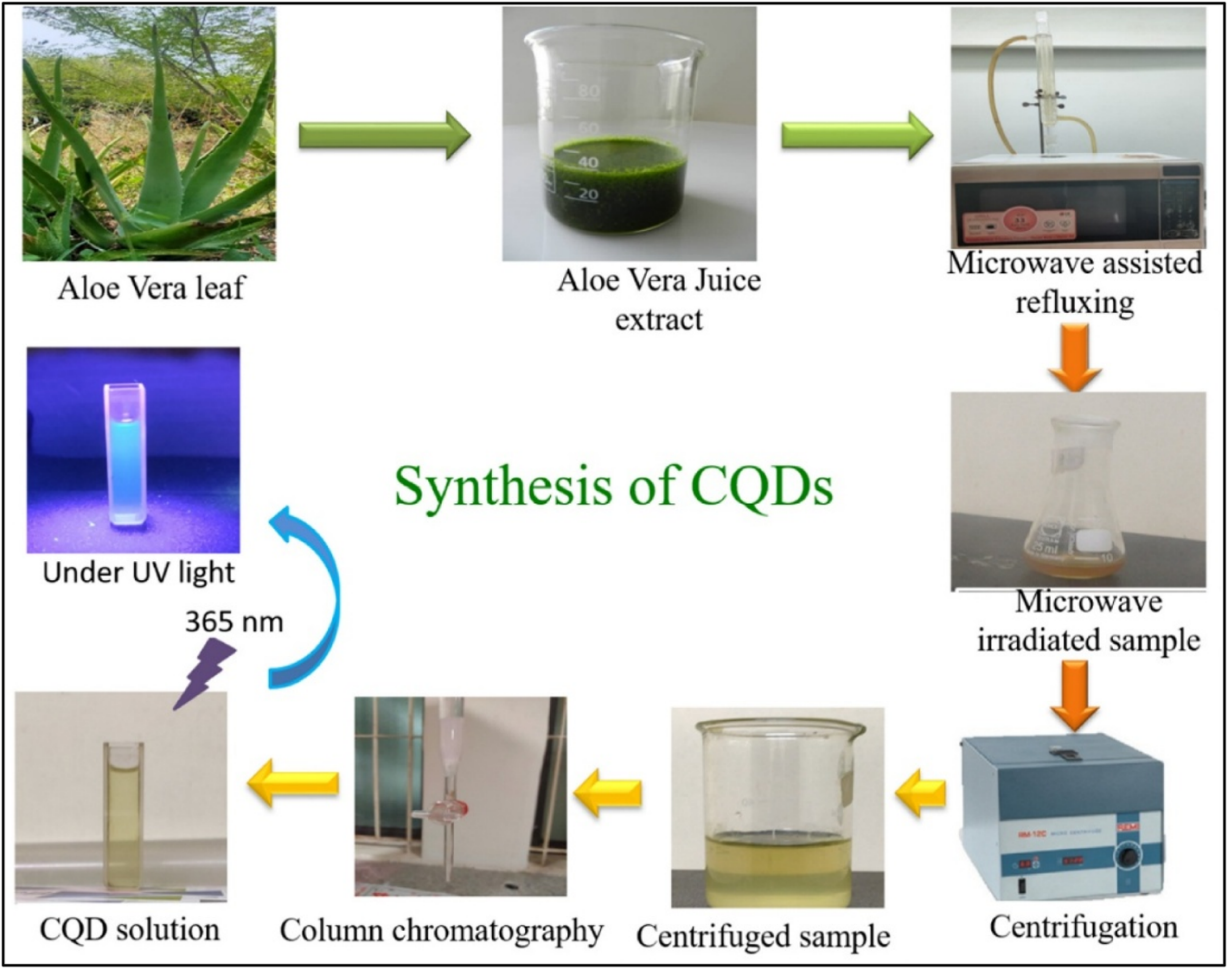
Schematic illustration of the preparation of CQDs from Aloe vera leaf extract. Source: reproduced with permission from Malavika *et al.* (2021).^49^

CQDs were also synthesized *via* a sustainable green approach with aloe vera extract, as represented in Fig. 3. Fresh leaves were processed, and the extract was subjected to microwave-assisted reflux at controlled power settings. After heating cycles, the solution was centrifuged and purified through silica gel column separation and dialysis, ensuring high-purity CQDs.^49^ CQDs were also synthesized from walnut oil *via* a hydrothermal technique. Walnut oil was mixed with water and heated in an autoclave at 180 °C. High-temperature conditions facilitate the breakdown of organic molecules into CQDs, which are then purified through centrifugation and filtration.^50^ A microwave-assisted green synthesis approach was employed for CQD production *via* Mesosphaerum suaveolens extract. The extract was subjected to microwave irradiation, facilitating organic compound breakdown into CQDs. The synthesized CQDs were isolated through centrifugation and filtration, ensuring a sustainable and cost-effective process.^51^ Similarly, fluorescent CQDs were synthesized from Mexican Mint (Plectranthus amboinicus) leaves *via* microwave-assisted reflux. The leaf extract was processed under controlled microwave conditions, followed by centrifugation and chromatography for purification. The final product was vacuum-dried to obtain high-purity CQDs.^52^ Finally, carbon dots (CDs) were synthesized from Piper longum leaves *via* a hydrothermal carbonization method. The leaves were ground, dispersed in water, and subjected to controlled heating in a hydrothermal autoclave. After carbonization, the solution was centrifuged and filtered, yielding high-quality aqueous carbon dots (PLACDs).^53^

### 3.3. Synthesis using biomass waste

The increasing demand for environmentally friendly nanomaterials has led to the exploration of biowaste as a potential precursor for synthesizing CQDs and other QDs. Various agricultural and food wastes, such as sugarcane bagasse, fruit peels, and human hair, have been successfully utilized for the synthesis of CQDs because of their high carbon content. Sugarcane bagasse, a fibrous residue from sugarcane processing, serves as a rich carbon precursor. The bagasse was thoroughly washed, air- and sun-dried, and then cut into small pieces before being subjected to hydrothermal treatment in a sealed container under high pressure and temperature. The organic compounds in SB underwent carbonization and dehydration, forming CQDs with optimized fluorescence properties.^54^ Similarly, sweet lemon peel, an agricultural waste, is subjected to hydrothermal treatment, where its natural polysaccharides act as reducing and stabilizing agents. These compounds facilitate carbonization and prevent aggregation, resulting in CQDs suitable for bioimaging and sensing applications.^55^

Plastic-derived CQDs were obtained from waste polyolefin residue *via* chemical oxidation combined with ultrasonic treatment in sulfuric acid and nitric acid to introduce oxygen-containing functional groups. The resulting suspension was then subjected to hydrothermal treatment at 120 °C, followed by neutralization and dialysis, yielding CQDs with green fluorescence, excellent aqueous stability, and a quantum yield of 4.84%.^56^ Human hair-derived CQDs were synthesized *via* microwave treatment at 180 °C, forming a black precipitate that was further sonicated and functionalized with poly-l-lysine (PLL) to enhance their properties.^57^ Watermelon peel extract, which is rich in phytochemicals, acts as a reducing agent for titanium chloride. The reaction was accelerated *via* microwave heating and ultrasonication, followed by centrifugation and lyophilization to obtain TiO_2_ QDs with excellent uniformity and stability.^58^ Similarly, kiwi fruit peels undergo hydrothermal treatment at 200 °C, where aqueous ammonia facilitates carbonization, resulting in a yellowish-brown supernatant containing CQDs with desirable optical properties, making them useful for nanotechnology applications.^59^

Furthermore, nitrogen-doped graphene quantum dots (N-GQDs) were synthesized using arjuna bark as a carbon source and a waste melamine sponge as a nitrogen source. The precursors were ultrasonicated, microwave-treated at 700 W for 10 min, centrifuged, filtered, and dialyzed, resulting in high-purity N-GQDs with enhanced optical and electronic properties.^60^ A similar hydrothermal method was employed for banana peel-derived nitrogen-doped CQDs (BP-CDs), where the peels were mixed with aqueous ammonia and heated at 200 °C for 24 hours, producing nanodots with a uniform size (∼5 nm), high photostability, and biocompatibility for bioimaging applications.^61^ Onion peel-derived CQDs were synthesized through boiling, stirring, and hydrothermal treatment with ethylene diamine (EDA) at 120 °C, which facilitated carbonization and functionalization, yielding highly fluorescent carbon dots.^62^ Citrus fruit peel-derived CQDs were obtained through a sand bath heating process at 180 °C for 12 hours, where organic compounds underwent dehydration and carbonization, producing brown-colored carbon dots that were purified *via* centrifugation and filtration.^63^ Likewise, palm oil empty fruit bunch (EFB)-derived CQDs were synthesized through hydrothermal carbonization at 180–220 °C, followed by centrifugation, filtration, and dialysis, leading to high-purity carbon dots with tailored optical and structural properties.^64^

### 3.4. Synthesis using biomacromolecules

Biomacromolecules have emerged as highly promising biotemplate or reducing agents in the green synthesis of QDs because of their intrinsic molecular recognition capabilities, biocompatibility, and structural diversity. Amino acids, the basic building blocks of proteins, are inexpensive, biocompatible, abundant and environmentally friendly. The availability of various functional groups, particularly both carboxyl and amino groups, renders them good molecular precursors for the programmable bioinspired synthesis of BQDs with projected characteristics derived from biomolecules.^65–67^ Remarkably, the nitrogen- and sulfur-rich functional groups present in amino acids also have the added benefit of doping biomolecule-sourced QDs (CQDs and GQDs) with heteroatoms, thereby leading to improved crystallinity and remarkable optical properties.^68,69^ In addition, the presence of highly efficient mixtures of various functional groups and amino acids provides improved programmability for the assembly of high-quality biomolecule-derived QDs. For example, C-QDs were fabricated through microwave irradiation of histidine under acid/alkali conditions, which displayed intense blue luminescence (with a QY of 44.9%) and increased chemiluminescence in the presence of a sodium periodate-hydrogen peroxide system.^70^ Glutamic acid has been utilized successfully as a carbon source for the synthesis of CQDs, providing a quantum efficiency of 30.7% with excitation-tunable fluorescence characteristics, which makes them suitable for use in plant cell imaging.^71^ Pyrolysis of l-glutamic acid has also resulted in the synthesis of highly stable and biocompatible GQDs with strong excitation-dependent luminescence and near-infrared (NIR) emission (800–850 nm), facilitating their use as *in vitro*/*in vivo* biomarkers and hydrogen peroxide sensors.^72^ Furthermore, nitrogen-doped GQDs functionalized with amino acids have been prepared with glycine, which emits blue fluorescence with a QY of 16.2%, and are used for highly sensitive fluorometric detection of Fe^3+^ ions.^73^

Proteins, as natural biopolymers, possess a rich variety of functional groups located along their peptide backbones, which can unfold into peptide chains under certain reactions and environmental conditions. This structural diversity, including groups such as thiols, carboxylates, and amines, renders proteins good multifunctional ligands. Consequently, they not only serve as templates or stabilizers in the synthesis of other metal-based quantum dots but also play important roles in the surface modification of preexisting hydrophobic QDs, making them more suitable for biological use and increasing their biomedical significance.^74–76^ A range of proteins, such as bovine serum albumin (BSA), β-lactoglobulin, hemoglobin, and gelatin, have been investigated as precursors for the production of biocompatible and highly fluorescent biomolecule-derived QDs. Of these, BSA is a native and denatured form that has been extensively applied as a green and easy precursor to produce CQDs. For example, hydrothermal treatment of BSA in the presence of a surface passivating agent has yielded blue-emitting, low-toxicity C-QDs for cellular imaging.^69^ Hemoglobin, made up of a heme group (with Fe^2+^ incorporated in a porphyrin ring) and four protein subunits, has also been used to synthesize blue-fluorescent C-QDs, popularly known as “blood dots,” which have been used as sensors for hydrogen peroxide.^74^ Analogously, gelatin has been used as a proteinous carbon source to synthesize C-QDs with blue luminescence, 31.6% quantum yield, and excitation dependent, upconversion, and pH-sensitive fluorescence—potentially useful in both bioimaging and fluorescent ink applications.^77^

Nucleic acids are biological polymers of natural origin made of reiterated nucleotide units, each having a nitrogenous base (purines or pyrimidines), a phosphate group, and a pentose sugar. Differences in structure among the pentose sugars and certain nucleobases permit division into two major types: RNA, with ribose (with hydroxyl groups) and the base uracil, and DNA, with deoxyribose (without one hydroxyl group) and the thymine base in place of uracil.^78^ Qiu and colleagues reported the successful synthesis of blue-emitting biodots through low-temperature hydrothermal processing of polycytosine DNA. These nanodots, when combined with Ag^+^ ions, were applied in the sensitive detection of biothiols and glutathione reductase activity.^78^ In another study, hydrothermal treatment of DNA yielded blue fluorescent CQDs, which proved effective in detecting mercury and silver ions in aqueous solutions.^79^Table 1 summarizes various biological methods used for the synthesis of bioinspired quantum dots.

**Table 1:** Overview of the properties and applications of bioinspired quantum dots synthesized from various sources

Sl no.	Source	QDs	Synthesis approach	Luminescence colour	Quantum yield	Applications	Ref
1	Lignin	GQDs	Hydrothermal	Blue	21	Photostability, biocompatibility	80
						Efficient nanoprobes for multicolour bioimaging	
2	Milk	CQDs	Hydrothermal	Blue	9.6	Prolonged lifetime and stronger excitation-dependency	81
						Improved energy transfer properties	
3	BBQ meat	CQDs	Thermal annealing	Green	40	Sustainably produced from abundant, renewable precursors, expanding their potential for diverse applications	82
4	Honey	GQDs	Carbonization	Green	3.6	Cost-effective synthesis method	83
						Suitable for industrial-scale production as biocompatible fluorescent inks for applications like anticounterfeiting	
5	Coffee grounds	CQDs	Simple heating	Blue	3.8	Cell imaging and surface-assisted laser desorption/ionization-mass spectrometry	84
6	Sugarcane molasses	CQDs	Thermal treatment	Blue	5.8	Successfully detected the food colorant sunset yellow (0–60 μM range) *via* fluorescence quenching	85
7	Cyanobacteria	CQDs	Ultrasound irradiation	—	—	Multifunctional T-tags enable targeted chemotherapeutic delivery to cancer cells while simultaneously activating G-tag fluorescence to monitor drug release in real time	86
8	Mushroom fungus	CQDs	Hydrothermal	Blue	15.3	Sensing applications for assaying HA and HAase	87
9	Bacterial genomic DNA	CQDs	Hydrothermal	Blue	—	Good fluorescent sensing property for Hg(ii) and Ag(i)	79
10	Gelatine	CQDs	Hydrothermal	Blue	31.6	Stable emission, well dispersibility, reduced toxicity, prolonged emission life time, and good compatibility	77
11	β-Lactoglobulin	CQDs	Hydrothermal	Blue	56	For the detection of different metal ions in a biological bioenvironment	88
12	Denatured BSA	CQDs	Microwave	Blue	14	Efficient upconversion fluorescent characteristics	89
13	Glycine	GQDs	Heating	Blue	16.2	Dosage-mediated selectivity toward Fe^3+^ amongst other metals	90
14	Isoleucine	CQDs	Hydrothermal	Blue	—	Sensitive and selective “turn-off” fluorescent probe for Fe^3+^ detection	91
15	Histidine	CQDs	Hydrothermal	Blue	8.9	Fe^3+^ sensing with a 10 nM detection limit, and biocompatibility, making them suitable for metal ion detection	92
16	Hyaluronic acid-glycine	CQDs	Autoclave	Blue	—	Strong colloidal stability, good biocompatibility, and selective uptake	93
						Promising cell-specific fluorescent probes for targeted tumor imaging and labelling	
17	Orange juice	CQDs	Hydrothermal	Green	26	High photostability and low toxicity serve as highly effective probes for cellular imaging applications	94
18	Banana juice	CQDs	Simple heating	Green	8.95	High-yield method that required no specialized equipment or reagents	95
19	Grape seeds	GQDs	Microwave	—	31.79	In selective organelle labelling, nucleus-targeted theranostics, and optical sensing probes	96
20	Watermelon peels	CQDs	Pyrolysis	Blue	7.1	Live cell imaging, demonstrating their potential as high-performance optical imaging probes	97

## 4. Types of bioinspired QDs

### 4.1. Carbon quantum dots

CQDs are carbon-based nanoplatforms that have received much interest because of their amazing optical, electrical, and chemical capabilities. CQDs are quasispherical nanomaterials with sizes less than 10 nm and exhibit unique features, such as photoluminescence, biocompatibility, and flexibility, allowing for extensive applications, including imaging, sensing, catalysis, and chemotherapeutic delivery. Traditional methods for CQD synthesis frequently use harsh chemicals and energy-intensive procedures, whereas bioinspired alternatives that use natural precursors such as plant extracts, biowaste, and microorganisms provide a more sustainable and environmentally friendly alternative. Bioinspired synthesis typically involves extracting bioactive chemicals, exposing them to mild carbonization, and purifying the resulting CQDs. These CQDs have a variety of applications in bioimaging, sensing, catalysis, and energy storage because of their intense photoluminescence and low toxicity. Despite its potential, issues such as scalability, reproducibility, and exact property control remain. Future research should concentrate on understanding synthesis mechanisms, standardizing protocols, and investigating novel applications in sectors such as flexible electronics and wearable devices. Bioinspired carbon quantum dots are a key step toward sustainable nanotechnology, minimizing environmental impact and opening the door to fresh scientific and industrial uses. Recent research has emphasized the potential applications of bioinspired CQDs in various sectors, including biomedicine and environmental monitoring. For example, the synthesis of CQDs from natural sources such as *Rosa indica* and *Citrus limetta* exemplifies a green strategy that not only uses abundant plant resources but also improves the luminous qualities of the produced nanoparticles. CQDs produced from *Citrus limetta* have been demonstrated to prevent *Candida albicans* biofilm development, highlighting their potential as antimicrobial agents in medicinal applications.^98^

**Fig. 4:**
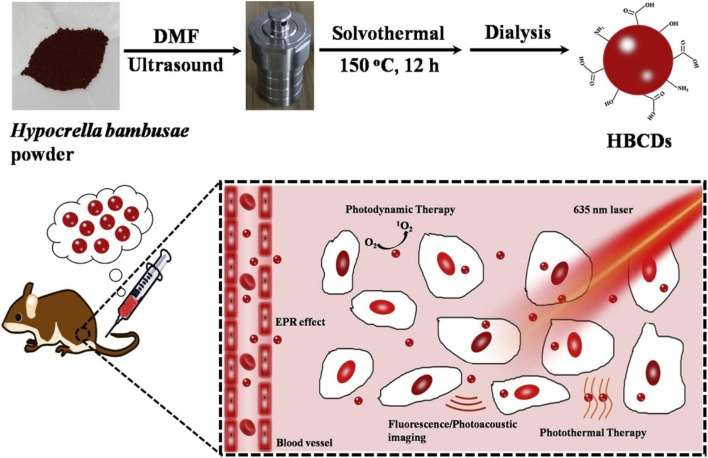
Schematic illustration of HBCDs derived from *hypocrella bambusae* for bimodel FL/PA imaging and synergistic PDT/PTT of cancer (Source: reproduced with permission from Jia *et al.*, 2018).^99^

Jia *et al.* synthesized theranostic, highly water-soluble carbon dots with broad light absorption from 350–800 nm and reduced biotoxicity from *Hypocrella bambusae* (HB) (Fig. 4). It is a parasitic fungus found in bamboo that is widely utilized in traditional Chinese medicine for the treatment of conditions such as rheumatoid arthritis, gastric disorders, and skin ailments. Extracts from HBs, known as hypocrellins, have demonstrated significant antiviral and anticancer properties through their photodynamic activity. The as-synthesized CDs exhibited efficient bimodal fluorescence/photoacoustic synergistic PDT and PTT.^99^ Similarly, CDs produced from oyster mushrooms have been significantly applied for colorimetric sensing of metal ions such as heavy metals. In addition, it has antibacterial activity and acts as a fluorescence probe for DNA detection.^100^ Ozdemir and coinvestigators synthesized versatile, bioinspired metal sulfide QD nanoplatforms for optoelectronic applications.^101^ Photoluminescent C dots were synthesized from ginsenoside Re *via* a facile hydrothermal method. Re-CDs exhibit efficient biocompatibility with efficient anticancer activity and bioimaging properties.^102^

Furthermore, the generation of highly fluorescent CQDs from almond resin *via* a one-pot microwave synthesis approach demonstrates their potential use in theranostics. These CQDs are highly biocompatible and photostable, making them viable alternatives to conventional synthetic dyes for live-cell imaging and diagnostics.^103^ The ability of bioinspired CQDs to be synthesized *via* multiple natural precursors emphasizes their versatility, which not only simplifies the production process but also decreases the environmental effect compared with standard synthetic approaches.^104^ In addition to their imaging ability, bioinspired CQDs have been investigated for their catalytic properties. Their surface functionalization can improve electron transport processes, making them efficient catalysts for a variety of chemical reactions.^104^ This multidimensional activity establishes bioinspired carbon quantum dots as a significant step forward in nanotechnology, paving the path for novel solutions in drug delivery, imaging, and environmental applications. Overall, the use of natural materials in the CQD synthesis process represents a sustainable strategy that is consistent with current trends toward eco-friendly nanomaterials.

### 4.2. Graphene quantum dots

Graphene quantum dots (GQDs) have recently gained wide consideration in cancer therapy because of their characteristic features, such as high biocompatibility, programmable photoluminescence, and minimal toxicity. These nanoscale materials, produced from graphene, have outstanding characteristics that make them suitable for use in targeted drug administration, imaging, and PTT.^105^ The bioinspired synthesis of GQDs frequently employs natural precursors sourced from plants or other biological sources, which increases not only their biocompatibility but also their functional properties. GQDs made from plant extracts, for example, have shown exceptional drug delivery and anticancer potential but remain low in toxicity. This green synthesis strategy is consistent with current trends in sustainable and ecologically friendly nanomaterials, providing a safer alternative to standard chemical processes.

Recent studies have revealed that GQDs can selectively target and treat cancer cells. For example, amine-functionalized GQDs, functionalized with nucleus-targeting peptides, have been shown to effectively penetrate the nuclei of cancer cells, inducing DNA damage and triggering apoptosis. This approach highlights the potential of GQDs as anticancer agents by directly disrupting the cellular mechanisms critical for tumor development.^106^ The bioinspired synthesis of GQDs frequently employs natural precursors sourced from plants or other biological sources, which increases not only their biocompatibility but also their functional properties. GQDs made from milk, for example, have shown excellent drug delivery and anticancer potential while being low in toxicity. This green synthesis strategy is consistent with current trends in sustainable and ecologically friendly nanomaterials, providing a safer alternative to standard chemical processes.^107,108^

A straightforward and effective method has been developed to produce GQDs from Miscanthus biowaste. This technique comprises rapid and precise removal of significant amounts of lignin and hemicellulose through acid hydrotrope fractionation, followed by a hydrothermal process. The resulting M-GQDs offer numerous benefits, including a single-crystalline, few-layer graphene-like structure, sulfur and nitrogen codoping, intense fluorescence, excitation-mediated photoluminescence, and a prolonged fluorescence duration of 11.95 ns.^109^ A similar study by Wang *et al.* synthesized N-GQDs from P. edulia *via* a single-step thermal process of green synthesis. The prepared N-GQDs exhibited efficient multicolor emission with effective Ag^+^ sensing and cell imaging properties.^110^

**Fig. 5:**
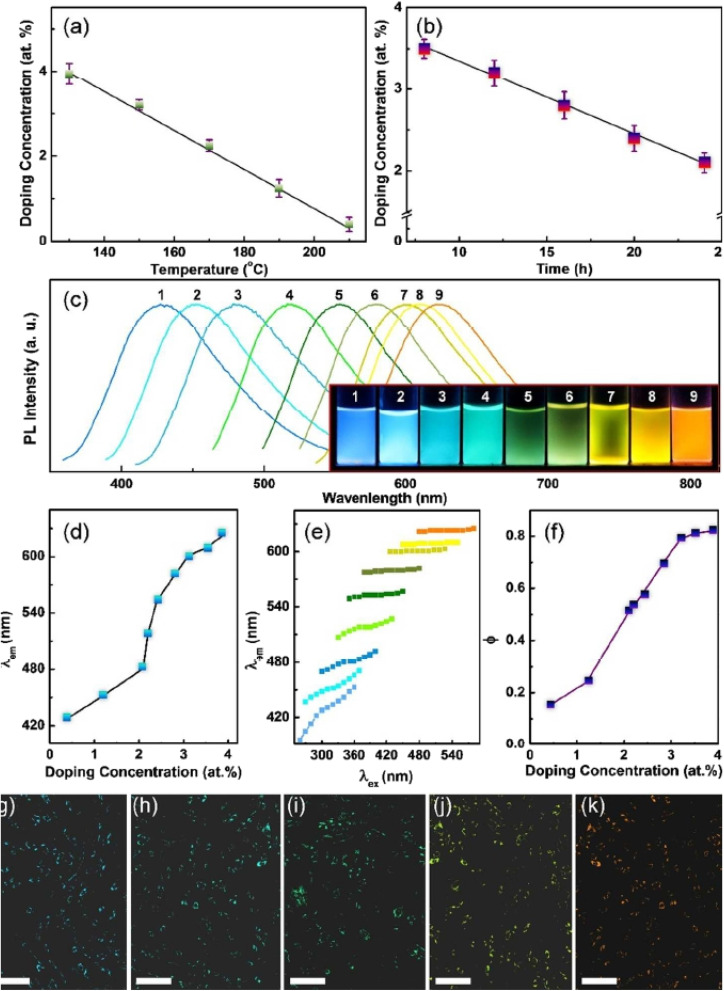
Control of the doping concentration of S-GQDs. Doping concentration of S-GQDs obtained at different (a) reaction temperatures and (b) reaction times. (c) PL spectra of S-GQDs with different doping concentrations. Inst: photographs of S-GQDs with different doping concentrations under 365 nm UV light. (d and e) Relationships between the doping concentration and (d) *λ*_ex_, (e) the excitation wavelength-dependent behavior and (f) the quantum yield of S-GQDs. (g–k) Confocal fluorescence microphotograph of fibroblasts (scale bar: 20 μm) incubated with S-GQDs, (g) S-GQDs-1, (h) S-GQDs-3, (i) S-GQDs-5, (j) S-GQDs-7 and (k) S-GQDs-9. (Source: reproduced with permission from Wang *et al.*, 2018).^113^

Chen *et al.* developed a green and effective one-pot hydrothermal approach for synthesizing GQDs from the natural polymer starch. The reactants for the synthesis are starch and water with no use of harsh chemicals such as strong acids or other oxidizers. The synthesis process is faster and requires approximately 2 h for completion. The prepared QDs were between 2.25 and 3.50 nm in diameter and involved hydrolysation and ring-closure condensation. This material exhibited satisfactory emission, reduced toxicity, the desired biocompatibility and improved aqueous solubility. The prepared GQDs were successfully employed for the bioimaging of cervical cancer.^111^ Nitrogen-doped QDs were synthesized from a cost-effective and nontoxic polymer, chitosan, *via* a chemical vapour deposition method. Chitosan, the second most prevalent biopolymer in nature, has gained wide consideration in recent decades because of its low cost, nontoxicity, biocompatibility, and versatility. The thermal decomposition of chitosan yields both carbon and nitrogen, which are used to create graphene QDs.^112^ Wang *et al.* developed a simple and efficient strategy for the synthesis of sulfur-doped GQDs *via* the hydrothermal process of durian using platinum as a catalyst. The proposed method exhibited a high quantum yield and demonstrated that the thiophene structure resulted in better optical and improved chemical stability. The prepared GQDs exhibited excellent photo/chemical stability and better photoluminescent efficiency (Fig. 5).^113^

### 4.3. Metallic QDs

The sustainability, biocompatibility, water solubility, increased luminosity, and variety of functions of BQDs, a recently discovered category of fluorescent bionanodots produced from abundant natural sources, have drawn much attention.^114^ QDs, occasionally referred to as semiconductor crystals, are actually a group of inorganic fluorophores that exhibit exceptional photophysical characteristics and are growing in application in industry and medical imaging.^115,116^ When compared to conventional medications, QDs show strong cancer growth suppression and precise cellular damage against cancer cells through their interaction with the phosphate groups on DNA.^117^ Additionally, they exhibit promise as tumor imaging agents because of their size-dependent photoluminescent characteristics.^118,119^ Multiple imaging technologies have become more popular because of their ability to accurately diagnose tumors and prevent physical stress.^120^ Transitional metals, such as manganese, zinc, lead and cadmium, can be conjugated with tellurides, selenides, sulfides and oxides to develop chalcogenide molecules or chalcogens. A bioconjugate comprising an antiestrogen alpha (ER alpha) antibody along with a cadmium sulfide-selenide (CdSSe) core in zinc sulfide (ZnS) shell quantum dots (core–shell) was created for the molecular detection of the breast cancer antigen ER alpha. This technique's precise assessment of ER alpha and high fluorescence resonance energy transfer (FRET) efficiency were made possible by improved biological conjugation along with FRET, suggesting a broad linear range for possible diagnostic applications.^121^

Leveraging the interaction of Herceptin with the HER-2 receptor located on the cell membrane, CdSe/ZnS core/shell QDs were coupled with Herceptin to specifically interact with HER2-overexpressing breast carcinoma cells (SK-BR3) and increase apoptosis. The possibility of their application for selective cancer therapy was highlighted by the evaluation of this interaction, which revealed that Herceptin-conjugated QDs predominantly targeted and caused cell death in SK-BR3 breast carcinoma cells while sparing normal or noncancerous cells (KBs).^122^ Since cadmium ions have the potential to gradually permeate the biological environment, several studies have examined the detrimental effects of cadmium-based QDs.^123–125^ The usage and disposal of cadmium-based nanoparticles raise serious environmental issues in addition to potential health impacts. The development of novel, cadmium-free QDs as nonhazardous and safe biological probes that retain these desirable optical characteristics and have potential clinical applications is currently a top priority because of these toxicity and environmental issues.^126–128^ Therefore, Mondel *et al.* demonstrated that (Zn)CuInS_2_ QDs having long fluorescence lifetimes that are biocompatible and devoid of cadmium are excellent bioimaging probes that improve the signal-to-noise ratio by a few orders of magnitude and decrease cell autofluorescence *via* time-gated-based detection. For the development of nonhazardous fluorescence imaging probes for highly sensitive biological diagnostics, these results are essential.^129^

Gold (Au) quantum dots are zero-dimensional formulations characterized by a small size, extensive surface area, and narrow conduction bands coupled with increased valency, which emit energy upon reverting to their ground state.^130^ The Au QD is made up of 5–25 Au atoms (less than 2 nm in size), absorbs ultraviolet (UV) rays and illuminates with fluorescence in the visible region caused by quantum effects. They also lack locally concentrated surface plasmon resonance activation.^131^ Owing to their absorption of near-UV light, the electrons present in the Au QDs are excited and undergo emission in the visible region. The length of luminescence is also dependent on the size (number) of the Au atoms in the QDs.^132^ Reticuloendothelial cells are the cells that nonspecifically absorb the majority of QDs.^133^ The 5–6 nm range is an approximate threshold size to avoid renal clearance.^134^ Even 72 hours after injection, the FeS QDs (3.0 nm)^135^ and ultrasmall Ag2 Te QDs (about 4.3 nm),^136^ along with cobalt sulfide QDs that are enzyme-like in nature (14.5 nm), accumulate 3.3% of the injected dose at the tumor site.^137^

**Fig. 6:**
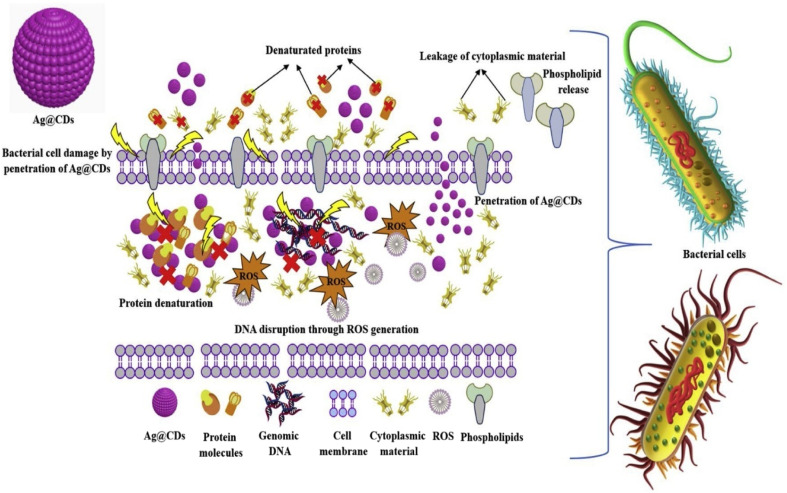
Illustration of the antibacterial mechanism of action of Ag@CDs *via* adsorption and subsequent penetration of Ag@CDs into the bacterial cell leading to cell wall deformation, protein denaturation, ROS generation, genomic DNA disruption, phospholipids release, and cytoplasmic leakage. (Source: reproduced with permission from Omran *et al.*, 2021).^145^

Nie *et al.* formulated a unique electrochemiluminescence (ECL) sensor utilizing a heterostructure of MXene-derived quantum dots (MQDs) and gold nanobones (Au NBs). First, the green chemistry technique was used to create MXenes and MQDs, avoiding the negative effects of hydrofluoric acid on both the environment and consumers. The MQDs conjugated with the Au NB heterostructure presented a significant increase in the ECL signal. Patients with triple-negative breast cancer had their serum levels of miRNA-26a measured *via* the MQD@Au NB-based heterostructure device for safe and efficient ECL sensing.^138^ However, the distribution pattern of BQDs can be altered by surface modification; for example, in a model of lung metastasis due to breast cancer, gold BQDs modified with macrophage-derived macrovesicles were incorporated into the lungs. *Cannabis sativa* leaf acetone extract was used for the phytosynthesis of CDs and Ag@CDs, whose antibacterial activity against *E. coli*, *S. aureus*, and the oral microflora was evaluated *via* agar well diffusion and microtiter plate assays (Fig. 6).^139^

Many studies have revealed the evident anticancer properties of silver nanoparticles (AgNPs) as radiosensitizing agents.^140–142^ In another study conducted by Esgandari *et al.*, the potential anticancer effects of radiation on breast cancer (BC) cells in conjunction with silver graphene quantum dots (SQDs) and 17-allylamino-17-demethoxy geldanamycin (17-AAG) were investigated. BC cell proliferation was inhibited, and apoptosis was induced through therapy with minimal quantities of 17-AAG along with SQD at a minimum hazardous concentration. Compared with the monotherapies, the triple combination significantly decreased cell viability, exhibiting lower levels of lactate. Therefore, when used in conjunction with 2 Gy of radiation as opposed to radiation monotherapy, it tends to enhance and augment the radiation effects to eventually produce anticancer results.^143^ The anticancer effects of novel zinc oxide quantum dots on breast cancer stem-like cells were studied, with particular emphasis on their effects on stemness markers, apoptosis, and cell proliferation. The biological activity of synthesized and described ZnO nanofluids was evaluated in mammospheres containing breast cancer cells. These findings revealed the potential of these nanoparticles as innovative therapies targeting breast cancer stem-like cells by showing that they might interfere with the JAK/STAT pathway, cause apoptosis, and decrease the expression of cancer stem cell markers.^144^

## 5. Surface functionalization strategies

Surface functionalization is essential to achieve the required functionality of BQDs. Biocompatibility is a critical requirement for the application of BQDs in biomedical investigations. However, most chemically synthesized QDs, particularly those produced in organic phases, face significant challenges due to their nonbiocompatibility, which restricts their usage in biomedicine. The primary issue arises from the hydrophobic organic ligands on the surface of QDs synthesized in organic phases, restricting their practical application in biological environments.

**Fig. 7:**
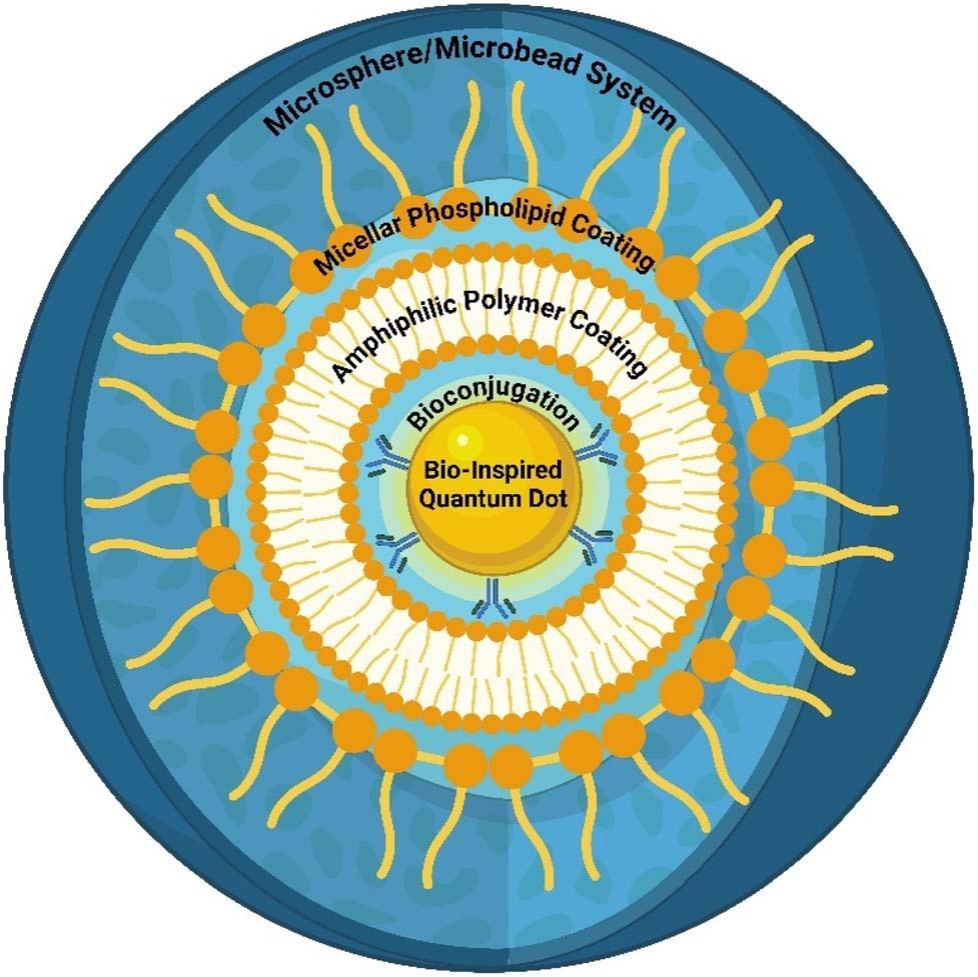
Surface functionalization approaches for bioinspired quantum dots.

To address this, approaches such as ligand exchange and encapsulation can be performed. Ligand exchange involves replacing the initial hydrophobic surfactant coating with a water-soluble stabilizer, whereas encapsulation involves modifying hydrophobic QDs with amphiphilic materials (Fig. 7). These approaches render hydrophobic QDs suitable for biomedical applications. The functional groups on BQDs can be partially controlled during synthesis by adjusting parameters such as the precursor type, doping elements, reaction temperature, pH, and time. BQDs synthesized from different biomass sources naturally possess surface groups such as –OH, –COOH, –NH_2_, and C=O, which can be enhanced or modified through heteroatom doping (*e.g.*, N, S, B). These groups are critical for targeting capabilities, as they enable further bioconjugation with ligands such as folic acid, peptides, or antibodies. Additionally, synthesizing QDs within biological or biomimetic systems can inherently enhance their biocompatibility. Biosynthesis methods, which are performed under mild conditions, can produce QDs with improved biocompatibility and stability, eliminating the need for further ligand exchange or encapsulation treatments.^32,146^ Functionalization with biomolecules such as proteins, antibodies, enzymes, and nucleic acids significantly enhances their cellular uptake and targeted biodistribution. These molecules offer multiple reactive groups (*e.g.*, amines, carboxyls, and phosphates) that enable stable conjugation with QDs, improving their targeting specificity, receptor-mediated internalization, and cellular retention.^147^

### 5.1. Salinization

With the growing demand for BQDs, there is an increasing emphasis on environmentally friendly synthesis methods. To reduce the toxicity associated with the use of harmful metal ions in QD preparation, salinization techniques are employed. For example, Bruna *et al.* (2019) demonstrated that Halobacillus sp. (DS2) could synthesize CdS QDs in the presence of high NaCl concentrations. This process is linked to the ability of bacteria to produce S^2−^ under such conditions. The purified biosynthesized QDs were characterized, and their stability was analysed across various NaCl concentrations.^148^ The presence of NaCl in biologically synthesized QDs is a novel characteristic, likely arising from the interaction between NaCl and biomolecules, such as peptides and proteins, that constitute the organic capping of the QDs.^149,150^

### 5.2. Encapsulation and ligand exchange approach

The presence of hydrophobic organic ligands on the surface of QDs makes their use in biological applications difficult. The two major approaches used to overcome these hurdles include encapsulation and ligand exchange methods, in which the surfactant coating of the QD is replaced with a hydrophilic stabilizer and amphiphilic molecules are replaced with QDs, respectively. These strategies make them appropriate for biomedical significance. The encapsulation strategy involves the functionalization of QDs with amphiphilic moieties containing functional groups such as –SH, –COOH, and –NH_2_ or with PEG or silica. The coating of these materials improved the dispersibility of the BQDs in water. Moreover, this approach surpasses the poor luminescent quantum yield of QDs, which usually aggregate or precipitate during surface functionalization. In a study by Yong *et al.*, phospholipid-modified CuInS_2_/ZnS QDs were prepared for efficient conjugation of folic acid for precise targeting.^151^ Silanization is a procedure in which the QD surface is coated with silica to improve its stability in biological media. The use of silica improves the biocompatibility of QDs and makes them suitable for *in vivo* applications. In addition, they are nontoxic, chemically inert in nature and safe to use. It is very easy to functionalize biomolecules such as antigens, proteins, aptamers and antibodies with silica, and they exhibit improved fluorescence and reduced aggregation when functionalized with monodispersed silica. In a reported method, silanization of CdZnSeS/ZnS QDs was performed *via* a water–oil microemulsion approach, where the QDs were small in size, exhibited reduced aggregation, improved luminescent quantum yield and good chemical and optical stability.^152,153^

The multidentate (MDT) ligand approach is another important surface functionalization approach in which a coordination complex is formed *via* the bonding of the carboxyl or amino groups of ligands with the central ions of QDs. MDT contains two or more atoms attached to a central atom. Curcurbituril is a widely used MDT that exhibits improved stability in complex biological environments and improved synthesis efficiency.^154,155^ However, it has several limitations, such as reduced luminescence intensity and activation energy. These limitations can be overcome by effectively regulating the reaction environment.^156^ Therefore, this functionalization allows QDs to become more hydrophilic and stable under biological conditions. Various QDs have been developed *via* solubilization processes, which involve capping QDs with functional moieties such as –NH_2_, –SH, or –COOH. This process of capping facilitates the controlled functionalization of these components. Likewise, aqueous-soluble QDs can be synthesized *via* aqueous-phase preparation methods, which employ water-soluble precursors.

### 5.3. Amphiphilic polymer coating

**Fig. 8:**
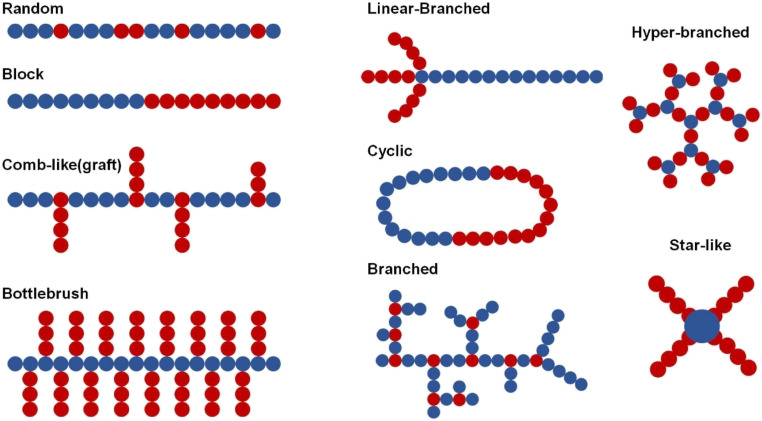
Typical molecular structures of amphiphilic copolymers for membrane applications (Source: reproduced with permission from Yi *et al.*, 2024).^158^

In biological settings, amphiphilic polymers are extensively recognized as highly adaptable stabilizing agents for hydrophobic QDs, guaranteeing their long-term colloidal stability and functionality in aqueous environments. These polymers have hydrophobic and hydrophilic domains. This makes them particularly suitable for modifying the surface characteristics of QDs and providing them with water–dispersible properties without compromising their intrinsic optical properties.^157^ Several amphiphilic polymers (Fig. 8), including polycaprolactone (PCL), poly(lactic-*co*-glycolic acid) (PLGA), and PEGylated poly(maleic anhydride-*alt*-1-octadecene) (PEG-PMAO), have been widely utilized for QD stabilization. PCL and PLGA, both FDA-approved biodegradable polymers, not only aid in QD dispersion but also provide controlled degradation profiles, making them ideal for drug delivery applications. Moreover, PEGylated PMAO combines hydrophobic stabilization with the antifouling properties of polyethylene glycol (PEG), significantly reducing nonspecific protein adsorption and prolonging the QD circulation time *in vivo*.^158^

The hydrophilic nature of amphiphilic polymer coatings is particularly advantageous in biological environments, as it significantly reduces nonspecific protein adsorption, a phenomenon known as opsonization. By resisting the binding of plasma proteins, these coatings prevent rapid clearance by the mononuclear phagocyte system (MPS), leading to an extended circulation half-life *in vivo*. This prolonged systemic retention enhances the likelihood of QD accumulation at tumor sites *via* the enhanced permeability and retention (EPR) effect—a passive targeting mechanism wherein nanoparticles preferentially accumulate in tumor tissues owing to leaky vasculature and impaired lymphatic drainage. The amphiphilic properties of the polymer coating further facilitate improved aqueous dispersibility, ensuring that the QDs maintain colloidal stability under physiological conditions without forming aggregates that can trigger immune recognition or clearance.^159^

Amphiphilic polymer coatings not only enhance the stability and biocompatibility of quantum dots but also provide a versatile platform for functionalization with various targeting ligands, enabling precise tumor-specific delivery. These polymers can be chemically modified to incorporate bioactive molecules such as folic acid, RGD peptides, transferrin, or monoclonal antibodies, which facilitate receptor-mediated endocytosis and improve cellular uptake in targeted cancer therapy. For instance, PEGylated QDs conjugated with anti-HER2 monoclonal antibodies specifically recognize and bind to HER2 receptors overexpressed on breast cancer cells, ensuring high affinity targeting. This specificity is particularly beneficial for both diagnostic imaging and targeted drug delivery, as it allows real-time fluorescence tracking of cancer progression while minimizing off-target effects and systemic toxicity.^160^

In addition to targeting ligands, amphiphilic polymer-coated QDs also serve as highly efficient nanocarriers for chemotherapeutic agents. The polymer matrix features a hydrophobic core, which provides an ideal microenvironment for encapsulating poorly water-soluble drugs such as paclitaxel, doxorubicin, or camptothecin. Upon reaching the tumor microenvironment, drug release can be triggered by various stimuli, such as pH-sensitive polymer degradation, enzymatic cleavage, or intracellular redox conditions, enabling a controlled and sustained drug release profile. This enhances drug accumulation at the tumor site while minimizing systemic exposure, thereby improving therapeutic efficacy and reducing dose-limiting side effects.^161^ In addition, these functionalized QDs can be engineered for multimodal applications, such as combining fluorescence imaging with drug delivery and photothermal or photodynamic therapy. The integration of additional functionalities, such as NIR dyes or photosensitizers, into amphiphilic polymer-coated QDs enables the use of synergistic treatments, where imaging, drug release, and light-induced tumor ablation occur simultaneously.^162^

Amphiphilic polymers, such as poly(ethylene glycol) (PEG)-based copolymers, enable the phase transfer of hydrophobic QDs synthesized in organic solvents into aqueous environments, ensuring their biocompatibility and stability under physiological conditions. This coating minimizes nonspecific interactions and enhances the specific binding of QDs conjugated with targeting agents, such as immunoglobulin G (IgG) or streptavidin, to biomarkers such as HER2 on breast cancer cells.^163^ Compared with organic dyes, the polymer coating also supports the retention of QDs' superior optical properties, including brighter fluorescence and higher photostability, facilitating the subcellular imaging of actin fibres, microtubules, and nuclear antigens. Moreover, the coatings enable dual or multimodal functionality, allowing the QDs to serve as both imaging agents and therapeutic carriers in breast cancer theranostics.^164^ In a study by Guang and colleagues, the amphiphilic polymer coating played a pivotal role in transforming hydrophobic CuInS2/ZnS quantum dots (QDs), which were initially coated with octadecylamine, into water-soluble, biocompatible probes suitable for breast cancer imaging. This centipede-like polymer encapsulation not only preserved the photoluminescence (PL) and ultraviolet–visible absorption properties of the QDs but also enabled their effective conjugation with anti-Ki-67 monoclonal antibodies to form QD-Ki-67 probes. This modification facilitated precise and specific labelling of Ki-67 in MDA-MB-231 cells, highlighting its importance in enabling QDs to function as effective biomarkers for breast cancer detection and prognosis.^165^

### 5.4. Micellar phospholipid coating

Micellar phospholipid coatings provide an effective strategy to increase the stability, functionality, and biocompatibility of QDs for breast cancer applications. These coatings rely on the self-assembly of amphiphilic phospholipids into micellar structures around the QDs, where the hydrophobic acyl chains interact with the QD surface *via* van der Waals forces, while the hydrophilic head groups extend outwards into the aqueous environment. This arrangement not only improves the colloidal stability of QDs in biological fluids but also prevents aggregation and undesired interactions with biomolecules.^166^

Commonly employed phospholipids include 1,2-distearoyl-*sn*-glycero-3-phosphoethanolamine (DSPE), which provides strong anchoring due to its long-chain hydrophobic tails; lecithin, a naturally occurring phospholipid known for its biocompatibility and emulsifying properties; and PEGylated phospholipids such as DSPE-PEG, which confer additional steric stabilization and prolonged circulation time *in vivo* by reducing opsonization and clearance *via* the reticuloendothelial system (RES). Furthermore, the functionalization of micellar phospholipid coatings with targeting ligands (*e.g.*, folic acid, peptides, or antibodies) enables the selective delivery of QDs to breast cancer cells, enhancing imaging contrast and therapeutic efficiency.^167^

The primary advantage of micellar phospholipid coatings lies in their biomimetic nature. By mimicking the lipid bilayer of cellular membranes, these coatings improve the compatibility of QDs with biological systems. For breast cancer management, such coatings have been used to encapsulate chemotherapeutic drugs such as doxorubicin or docetaxel within the micellar structure. This design enables the codelivery of imaging agents (QDs) and therapeutic agents, creating a robust platform for theranostic applications.^168^ Phospholipid-coated QDs can be functionalized with targeting molecules to achieve site-specific delivery. For example, conjugation with aptamers or antibodies targeting CD44, a receptor overexpressed in triple-negative breast cancer (TNBC), enhances the selective uptake of QDs by cancer cells. Once internalized, the micellar coating ensures controlled drug release, which can be triggered by tumor-specific stimuli such as low pH, elevated reactive oxygen species (ROS), or enzymatic activity.^169^

Additionally, these coatings provide a means for reducing the immunogenicity and nonspecific binding of QDs. The PEGylated head groups create a steric barrier, further extending the circulation time of the QDs *in vivo*. Importantly, micellar phospholipid coatings protect the core of the QDs from oxidative and enzymatic degradation, ensuring sustained fluorescence for diagnostic imaging.^170^ In a study conducted by Benoit and colleagues, QDs were encapsulated within phospholipid block copolymer micelles, forming highly stable, water-dispersible, and biocompatible nanocrystals suitable for biological applications. The exceptional stability, low toxicity, and superior fluorescence retention of these QD-micelles highlight their potential for a wide range of biomedical imaging applications. Their ability to serve as long-term, highly specific molecular probes could revolutionize areas such as single-cell tracking, developmental biology, and targeted molecular diagnostics. Additionally, their robust photophysical properties make them promising candidates for next-generation bioimaging tools in both fundamental research and clinical applications.^171^

A study by Rui and colleagues highlighted the encapsulation of near-infrared-emitting lead sulfide (PbS) QDs in PEGylated phospholipid micelles for *in vitro* and *in vivo* imaging. Encapsulation reduces the toxicity of PbS QDs while maintaining their optical properties. The formulation exhibited robust cellular uptake in macrophages and pancreatic cancer cells, with folic acid functionalization enhancing targeted delivery. *In vivo* studies in mice demonstrated efficient accumulation in the liver and spleen with minimal acute toxicity.^172^ NIR whole-body imaging enables noninvasive visualization of tumors and biological processes. This study highlights poly(ethylene glycol)-phospholipid micelle-encapsulated quantum dots (QD-Ms) as superior contrast agents compared with PEGylated quantum dots (QD-PEGs). The QD-Ms achieved faster tumor accumulation (within 1 hour), higher signal–to–noise ratios (SNRs), and better imaging efficiency at half the dose of QD-PEGs. The QD-Ms provided sharper tumor and organ visualization, with minimal variability and stable biodistribution over time. They localized fluorescence effectively, while the QD-PEGs showed diffuse retention. Overall, QD-Ms offer enhanced imaging quality, faster results, and reliable quantification, making them promising tools for tumor imaging.^173^

### 5.5. Microsphere/microbead systems

Microspheres and microbeads serve as highly efficient microscale carriers that provide a protective and functional matrix for QDs, increasing their stability, bioavailability, and targeted delivery in breast cancer diagnosis and therapy.^174^ The integration of QDs into microspheres offers several advantages in breast cancer management. First, microspheres provide a physical barrier that prevents QD aggregation, degradation, or leaching under physiological conditions, thereby enhancing their photostability and fluorescence retention over extended periods. This stability is crucial for long-term imaging applications, where consistent signal intensity is required for tracking tumor progression. Second, microspheres allow for the controlled and sustained release of therapeutic agents coencapsulated with QDs, optimizing drug bioavailability at the tumor site. For example, PLGA microspheres coloaded with QDs and tamoxifen, a selective estrogen receptor modulator (SERM), can selectively deliver the drug to estrogen receptor-positive (ER+) breast cancer cells while simultaneously enabling fluorescence-guided imaging to monitor drug distribution and therapeutic response in real time.^160^

The microsphere matrix can be engineered to respond to tumor-specific stimuli, such as acidic pH or matrix metalloproteinase (MMP) activity, which triggers the release of QDs and drugs in the tumor microenvironment. Furthermore, the size and surface charge of microspheres can be tailored to maximize their accumulation in tumor tissues *via* the EPR effect. Microbead systems are particularly useful for multiplexed diagnostics. By labelling microbeads with QDs of different emission spectra, researchers can simultaneously detect multiple biomarkers in breast cancer tissues. This capability is valuable for profiling heterogeneous tumors and designing personalized therapeutic regimens.^175^ The functionalization of Y_2_O_3_ microspheres with BQDs offers a promising approach for breast cancer therapy by combining imaging and therapeutic capabilities. In this study, QDs were immobilized onto microspheres through dehydration–condensation reactions, imparting photoluminescence (PL) properties for high-sensitivity tracking in tissues. The optimal reaction conditions (≤6 hours) preserved the PL intensity and minimized QD aggregation, ensuring stability and precise imaging. Microspheres produced at 3000 rpm were optimal for cancer embolization, showing consistent size and surface properties for effective QD binding. The ability to tune PL emission wavelengths further enhances imaging applications. This functionalization strategy enables minimally invasive, theranostic solutions for breast cancer, improving tumor visualization and therapeutic precision.^176^

Furthermore, microsphere-based QD carriers can be functionalized with targeting ligands such as monoclonal antibodies, aptamers, or peptides, increasing their specificity toward breast cancer biomarkers such as HER2, EGFR, or folate receptors. This targeted approach minimizes systemic toxicity and off-target accumulation, improving therapeutic efficacy while reducing adverse effects. Additionally, the biodegradable nature of polymeric microspheres ensures that they degrade into nontoxic metabolites, making them suitable for clinical translation. Overall, the combination of QDs with microsphere-based delivery systems represents a promising strategy for advancing breast cancer diagnostics and therapy. These multifunctional platforms offer a synergistic approach to tumor imaging, targeted drug delivery, and therapeutic monitoring, paving the way for the development of next-generation nanomedicine-based precision oncology tools.^177^

### 5.6. Bioconjugation

Bioconjugation represents a cornerstone strategy for enhancing the specificity, targeting ability, and overall functionality of BQDs in breast cancer detection, imaging, and therapy. This process involves the covalent or noncovalent attachment of biologically relevant molecules—such as monoclonal antibodies, peptides, proteins, aptamers, nucleic acids, or small-molecule ligands—to the surface of QDs. By facilitating molecular recognition and selective interactions with cancer-associated biomarkers, bioconjugation significantly improves the targeting efficiency and diagnostic precision of QD-based nanoprobes.^178^

The functionalization of QDs is achieved through various chemical conjugation strategies, ensuring stable and bioactive linkages between the QD surface and the targeted biomolecule. Common crosslinking chemistries include carbodiimide-mediated coupling (*e.g.*, 1-ethyl-3-(3-dimethylaminopropyl) carbodiimide hydrochloride (EDC) and *N*-hydroxysuccinimide (NHS)), which facilitates the formation of stable amide bonds between carboxyl-functionalized QDs and amine-containing biomolecules. Click chemistry, particularly copper(i)-catalyzed azide–alkyne cycloaddition (CuAAC) and strain–promoted azide–alkyne cycloaddition (SPAAC), provides bioorthogonal and highly efficient conjugation, minimizing nonspecific interactions and maintaining biological integrity. Additionally, maleimide-thiol coupling is frequently employed to link thiol (–SH)–containing biomolecules to maleimide-functionalized QDs, ensuring site-specific and stable attachment.^179^ In addition to chemical conjugation, noncovalent strategies such as electrostatic interactions, hydrophobic interactions, and biotin-streptavidin binding offer alternative approaches for QD functionalization while preserving biomolecular activity. These bioconjugation techniques enable the development of highly specific QD-based probes for fluorescence imaging, targeted drug delivery, and real-time tracking of breast cancer cells, ultimately improving early detection, treatment efficacy, and personalized therapeutic strategies.

**Fig. 9:**
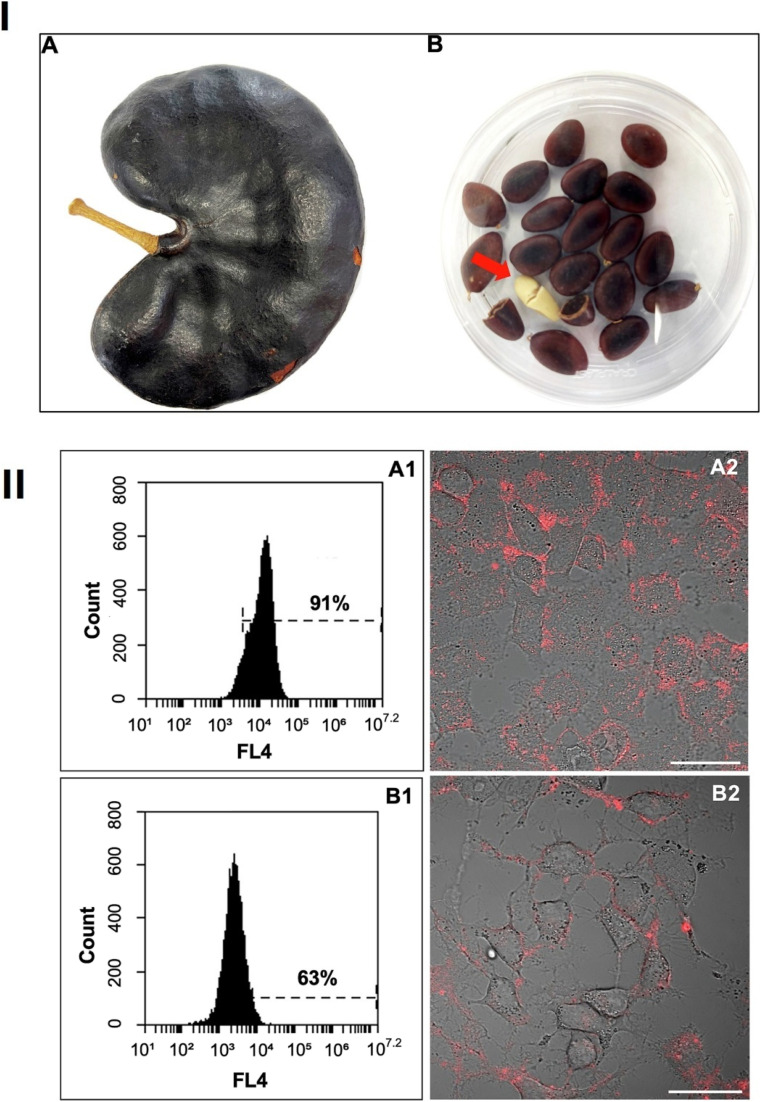
(I) Morphology of the fruit parts of *Enterolobium contortisiliquum*. (A): entire fruit and (B): cotyledons and a seed indicated by the red arrow. (II) Morphology of the fruit parts of *Enterolobium contortisiliquum*. (A): entire fruit and (B): cotyledons and a seed indicated by the red arrow. (Source: reproduced with permission from Santos *et al.*, 2023).^180^

The conjugation of *Enterolobium contortisiliquum* trypsin inhibitor (EcTI) with QDs *via* EDC/NHS chemistry represents a strategic approach for targeted breast cancer research. By activating carboxyl groups on QDs, EDC (1-ethyl-3-(3-dimethylaminopropyl)carbodiimide) and NHS (*N*-hydroxysuccinimide) facilitate stable covalent bonding, forming reactive NHS esters that efficiently bind to primary amines on EcTI. This ensures a stable amide linkage, preserving the protease inhibitory activity of EcTI. In breast cancer, particularly in MDA-MB-231 cells, QD-EcTI conjugates enable fluorescence-based tracking of serine protease interactions, highlighting differential protease expression. This targeted approach enhances imaging precision and could provide insights into protease-driven tumor progression, potentially guiding novel therapeutic strategies (Fig. 9).^180^ In the context of breast cancer, bioconjugation transforms QDs into highly specific imaging and therapeutic agents. For example, QDs conjugated with anti-HER2 antibodies can selectively bind to HER2-positive cancer cells, enabling high-resolution imaging and targeted drug delivery.^181^ Similarly, peptides such as arginine-glycine-aspartate (RGD), which targets integrins that are overexpressed in TNBC, can be conjugated to QDs for tumor-specific accumulation.^182^ Copper indium zinc sulfide (CuInZn_*x*_S_2+*x*_, ZCIS) QDs were surface-functionalized with the anti-HER2 peptide LTVSPWY, which is specifically designed to target HER2-overexpressing SKBR3 breast cancer cells. This targeted approach enables selective cellular uptake and high-affinity binding to HER2-positive tumors, allowing for precise imaging and enhanced therapeutic delivery.^183^

## 6. Applications of bioinspired QDs for breast cancer treatment

**Fig. 10:**
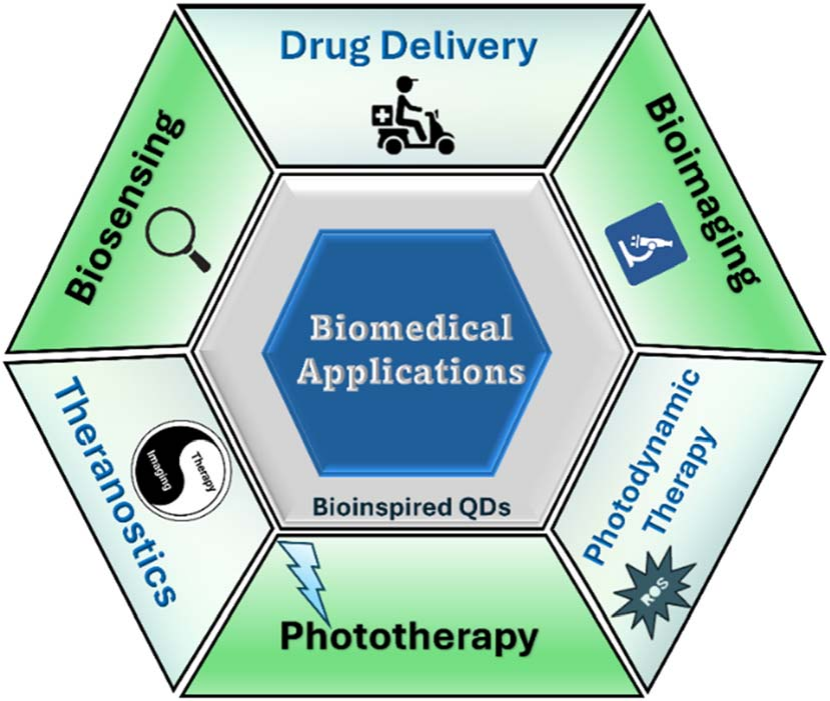
Diagrammatic representation of the applications of bioinspired QDs in breast cancer theranostics.

BQDs, which are generated from natural sources such as plant residues and biopolymers, provide a long-term and friendly alternative for breast cancer detection and treatment (Fig. 10). These QDs have remarkable fluorescence characteristics, allowing for precise imaging of cancer cells *via* tumor-specific ligands or antibodies. They also act as nanocarriers for targeted drug delivery, reducing toxicity while improving therapeutic outcomes, and their photodynamic properties cause cancer cells to die when exposed to light. Furthermore, bioinspired QDs act as biosensors, detecting cancer biomarkers and offering speedy and cost-effective diagnostics. Their natural origin decreases their degree of cytotoxicity, making them appropriate for therapeutic use. Despite scalability and regulatory obstacles, its integration with sophisticated technologies has the potential to improve breast cancer treatment. BQDs achieve selective targeting of cancer cells through both passive accumulation (*via* the EPR effect) and active targeting *via* ligand functionalization. A unique advantage of BQDs is their ability to facilitate single-ligand-mediated selective targeting, which is often challenging with conventional nanocarriers. For example, cysteamine hydrochloride-functionalized graphene quantum dots (Cys@GQDs) derived from milk selectively targeted MDA-MB-231 breast cancer cells over L929 and HeLa cells, likely due to enhanced cellular uptake and receptor-mediated interactions. This functionalization not only improved the selective cytotoxicity but also enhanced the delivery and efficacy of coloaded drugs such as berberine. These results highlight that surface engineering with a specific ligand can significantly increase the tumor selectivity and theranostic potential of BQDs.^108,184^

### 6.1. Biosensing and imaging

The characteristic optical and biocompatibility properties of BQDs have been explored in tumor diagnosis and bioimaging. The biomaterials obtained from natural materials exhibit improved fluorescence, functionalization abilities, and resistance to photobleaching, which makes QDs ideal for tumor diagnosis and imaging. The conjugation of QDs with breast cancer biomarkers such as antibodies and peptides results in the specific targeted imaging of breast cancer cells. The size-tunable fluorescence property of bioinspired QDs permits their use in the simultaneous sensing of various targets necessary for understanding cancer heterogeneity. In addition to their use in diagnostics, QDs help in real-time imaging of the cancer microenvironment, which helps in the real-time understanding of tumor invasion and metastasis. BQDs hold great promise for *in vivo* imaging because of their biocompatibility, aqueous solubility, and photoluminescent properties, yet several limitations persist. The use of Gd-doping for MRI-fluorescence dual imaging and NIR-absorbing BQDs for deeper tissue penetration and photothermal imaging is emerging and requires further optimization. The surface functionalization of QDs enhances the signalling of precise targeted therapy with efficacious drug delivery and reduced adverse effects. Despite their potential significance, issues such as cytotoxicity and nonspecific cellular uptake must be addressed before they can be used in more clinical applications. Ongoing studies are striving to improve QD development and function to improve cancer diagnosis and therapies. Doping BQDs with elements such as nitrogen, sulfur, or phosphorus alters their electronic structure and bandgap, increasing their ability to redshift or blueshift their emission. For example, nitrogen and boron codoping in graphene QDs enables NIR-II emission beyond 1000 nm.^29,185^

**Fig. 11:**
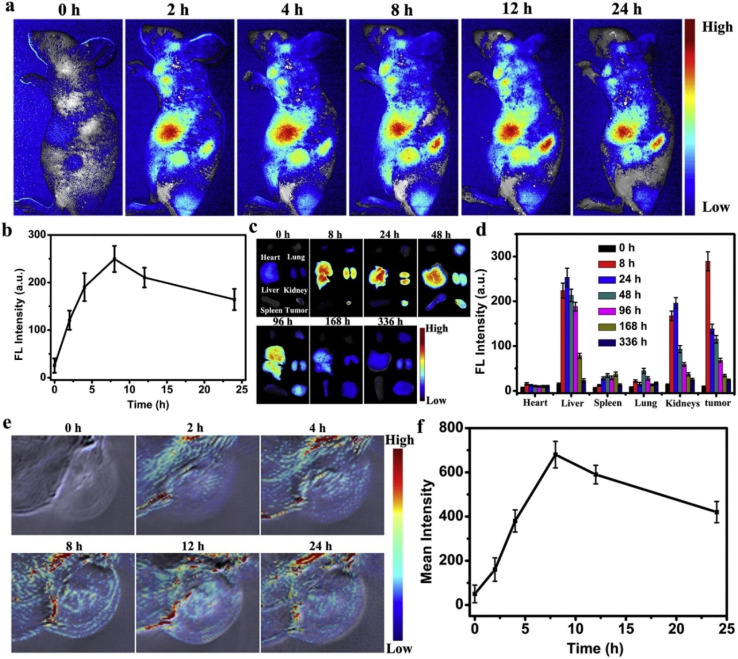
(a) *In vivo* FL imaging of mice post-*i.v.* injection of HBCDs in PBS. (b) FL intensities of tumors in (a). (c) *Ex vivo* FL imaging of tumor and major organs at different time points post-*i.v.* injection of HBCDs in PBS. (d) FL intensities of major organs and tumor in (c). (e) *In vivo* PA imaging of mice post-*i.v.* injection of HBCDs in PBS. (f) PA intensities of tumors in (e). Data are expressed as means ± s.d. (*n* = 3). (Source: reproduced with permission from Jia *et al.*, 2018).^99^

Jia *et al.* developed C dots from *Hypocrella bambusae* for fluorescence (FL) and photoacoustic (PA) imaging-mediated PDT and PTT of cancer. The photothermal activity of the CDs was evaluated by irradiating the solution with a 635 IR laser at 0.8 W cm^−2^, and a significant increase of 26.9 °C was observed, with a good PCE value of 27.6%. The *in vivo* biodistribution of the CDs was evaluated *via* FL/PA imaging, which revealed a maximum FL signal at 8 h (Fig. 11).^99^ In a similar study by Vandarkuzhali *et al.*, fluorescent CDs with relatively high fluorescence from the pseudostems of banana plants were prepared *via* a simple solvothermal approach for bioimaging and nanosensor applications. This study demonstrated efficient Fe^3+^ detection and cellular imaging. The as-synthesized CDs were pale yellow in color, and the optical characteristics of the prepared CDs were assessed *via* UV-vis studies, with absorption at 284 nm. Visualization of the CD solution *via* the naked eye makes it a suitable agent for nanosensing. The fluorescence spectra of the CDs showed excitation at 340 nm with increased resistance to increased ionic concentrations. Efficient photostability, increased fluorescence emission at physiological pH and increased ionic concentrations make these materials potential candidates for bioimaging and biosensing activities.^186^

**Fig. 12:**
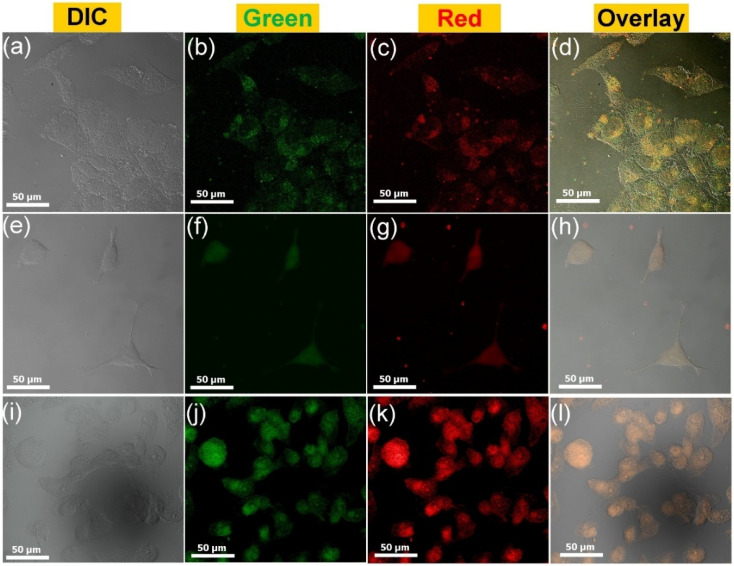
CLSM images at two laser excitation wavelengths: 488 nm (green) and 550 nm (red) using GQDs@Cys-BHC complex (final concentration; ∼200 μg mL^−1^ GQD concentration in the complex). Images show HeLa cells (a–d), L929 cells (e–h), and MDA-MB-231 cells (i–l) stained with GQDs@Cys-BHC complex. Cellular intake is clearly demonstrated by co-localization merged channels (Source: reproduced with permission from Thakur *et al.*, 2016).^108^

Milk-derived highly fluorescent bioinspired GQDs have significant biocompatibility and act as efficient bioimaging probes for cancer cells at relatively low concentrations, as shown in Fig. 12. The pH-mediated FL intensity increased with increasing pH from 1–6 and decreased drastically from pH 70–14. The CLSM technique was employed to evaluate the cellular localization of GQDs and exhibited fluorescence imaging under normal light and different excitation wavelengths.^108^ Bioinspired cadmium chalcogenide QDs prepared with the fungus *Rhizopus stolonifer* presented greenish blue and purple luminescence upon UV light exposure. The biosynthesized GQDs showed strong photoluminescence spectra with broadening, indicating their size distribution.^187^ GQDs prepared from the biopolymer starch *via* a facile one-pot hydrothermal approach were used for efficient bioimaging of cancer.^111^ Lin *et al.* prepared an albumin biomineralization strategy for the synthesis of cobalt sulfide NDs for multimodal imaging of cancer. Real-time analysis of phototherapy is useful for observing temperature changes because of its strong NIR absorption properties.^188^

Linseed is a nutritional, nontoxic material that is less expensive than other carbon precursors, such as soy milk, eggs and juice. Song *et al.* prepared CDs with efficient PL properties for bioimaging and biosensing applications. The as-synthesized CDs were utilized for MCF-7 cell bioimaging. The excitation-mediated PL property of the CDs was observed by the emission of green fluorescence upon irradiation with a 405 nm laser. In addition, CDs have been explored for biosensing in the quantitative evaluation of the butyrylcholinesterase enzyme.^189^ Ramanan *et al.* prepared fluorescent CDs from algal blooms for *in vitro* imaging of MCF-7 cancer cells. The prepared CDs were biocompatible and 8 nm in size, making them suitable for bioimaging. The intense green PL on the MCF-7 cell membrane and in the cytoplasmic area indicates the efficient penetration of the CDs.^190^ Yao *et al.* synthesized ginsenoside Rebased CDs with a diameter of approximately 4.6 nm with efficient PL properties for biosensing and desired anticancer activity.^102^ Sulfur- and nitrogen-doped CDs were prepared with *Allium fistulosum* for multicolor imaging of MCF-7 cells. The prepared CDs exhibited excellent biocompatibility and efficient thermal stability, making them suitable for nanoimaging.^191^

### 6.2. Chemotherapeutic delivery

BQDs are emerging as new medication delivery approaches, particularly in the field of cancer treatment. One of the key applications of BQDs is their potential to encapsulate therapeutic substances, such as anticancer medicines, and allow for tailored release at tumor locations. Targeted drug delivery is a novel therapeutic approach designed for the transportation of therapeutic moieties to specific locations, thereby improving therapeutic activity and reducing systemic adverse events. Various nanoplatforms have been explored in this field, and BQDs have emerged as versatile tools because they exhibit biocompatibility, characteristic optical properties and tunable surface functionalization. Owing to the quantum confinement property of QDs, they exhibit size-mediated electronic and optical features, which help in targeted drug delivery. The functionalization of QDs with peptides, antibodies or small molecules allows them to recognize specific biomarkers on the targeted cell. Compared with conventional QDs, BQDs functionalized with a ligand are stable against photobleaching and have reduced toxicity. In addition, these systems mimic biological processes, improving the biodistribution and targeting abilities of therapeutic agents. They exhibit common mechanisms, such as receptor-mediated endocytosis, controlled drug release, and use as theranostic agents (which exhibit both therapeutic and imaging properties). Specific targeting can be achieved in the case of target-specific breast cancers, such as ER- or FR-positive cancers, by incorporating specific ligands onto QDs.

**Fig. 13:**
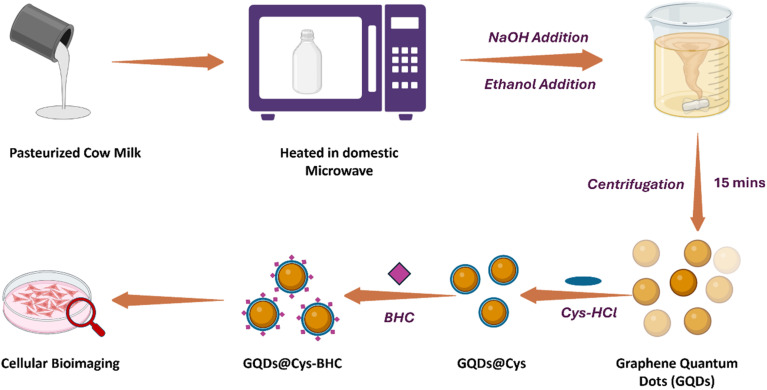
Diagrammatic illustration of graphene quantum dot (GQD) synthesis from pasteurized cow milk, functionalization with Cys-HCl, drug loading with BHCs, and application in cellular bioimaging.

Thakur *et al.* prepared multifluorescent graphene QDs from milk *via* an economical green-chemistry technique, as displayed in Fig. 13. The prepared QDs were then loaded with the anticancer drug berberine hydrochloride (BHC) for image-guided targeted cancer therapy. The drug-loaded QDs demonstrated a loading efficiency of 88% with pH-sensitive drug release. The results of the cytotoxicity studies performed on the MDA-MB-231 cell line revealed that the nanoformulation loaded with BHC has greater toxicity than the bare BHC does. This significant change could be attributed to changes in cellular uptake mechanisms and microenvironments.^108^ In another study by Sawant and coworkers, carbon dots were developed from ginger and coated with TiO_2_ NPs, followed by loading with curcumin. The developed nanoformulation exhibited an efficient loading percentage of 89%, with more release occurring at acidic pH values. The curcumin-loaded formulation exhibited improved antitumour activity in MCF-7 cells with enhanced biocompatibility.^192^ Similarly, biomimetic macrophage membrane-functionalized CDs and gold QDs loaded with DOX showed a loading efficiency of approximately 60%, with targeted antitumour activity in 4T1 cells.^193^

### 6.3. Phototherapy

BQDs are emerging as efficient candidates for PTT owing to their characteristic optical properties, photostability, tunable absorption spectra, versatility and biocompatibility. These nanoplatforms effectively absorb NIR light with deeper penetration into cancer tissues and negligible absorption by biological and water molecules. Upon irradiation with NIR light, QDs convert absorbed photons to heat, which causes hyperthermia in cancer cells and destroys them with minimal damage to surrounding healthy tissues. In addition, the small size of QDs allows them to penetrate efficiently into tissues with improved cellular uptake, which is ideal for targeted cancer therapy. BQDs can be engineered to increase their PCE by modulating their size, surface chemistry, elemental doping, and structural composition. Smaller BQDs with higher sp^2^ carbon contents and well-passivated surfaces tend to exhibit greater photothermal stability and effective tumor ablation with minimal off-target effects. These engineered features make BQDs promising agents for photothermal cancer therapy, especially when combined with imaging or drug delivery functionalities.^194,195^

The combination of PTT with other modalities, such as chemotherapy or immunotherapy, further potentiates therapeutic efficacy and escalates patient outcomes.^196,197^ The PCE of BQDs, similar to that of other carbon-based quantum dots (CDs), is strongly influenced by both their structural features and the laser irradiation parameters applied. QDs doped with both nitrogen and oxygen typically achieve a PCE > 50%, significantly outperforming undoped or singly doped counterparts (PCE < 30%). However, comparisons of these effects quantitatively are currently limited by incomplete data on uniform characterization parameters.^198^ QDs derived from natural or renewable sources demonstrate excellent photostability during prolonged imaging. Compared with many organic dyes or traditional semiconductor QDs, their unique carbon-based structures and electronic properties make them highly resistant to photobleaching and signal degradation under continuous excitation. These QDs maintain strong, consistent fluorescence even during long-term imaging, enabling reliable real-time and dynamic tracking of biological processes. Additionally, surface functionalization can further increase their stability by preventing aggregation and providing protection against oxidation or chemical interactions.^184,199,200^

**Fig. 14:**
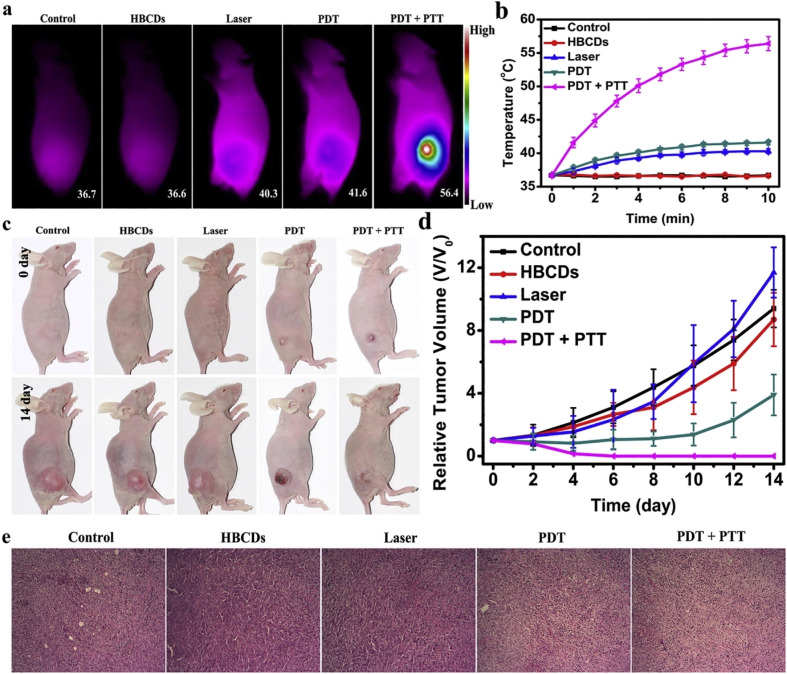
(a) IR thermal images of mice post different treatments. (b) Temperature curves of tumor during the irradiation. (c) Photographs of mice post different treatments. (d) The growth curves of tumor during different treatments. (e) H&E-stained slices of tumor post different treatments. Data are expressed as means ± s.d. (*n* = 5). (Source: reproduced with permission from Jia *et al.*, 2018).^99^

Jia *et al.* prepared phototheranostic CDs from *Hypocrella bambusae*, which exhibited the desired aqueous solubility and efficient light absorption. The photothermal activity of the prepared CDs was evaluated by irradiating the solution with 0.8 W cm^−2^ of 635 nm radiation. After 10 min of irradiation, the temperature increased to 26.9 °C, whereas the temperature of the water increased to only 5.4 °C. Furthermore, the thermograms further confirmed the ability of the CDs to efficiently convert light to heat. The photothermal conversion efficiency (PCE) of the prepared CDs was similar to that of other established photothermal agents (27.6%). The *in vivo* phototherapeutic activity of the prepared CDs was evaluated in a nude mouse tumor model (Fig. 14), which revealed efficient cancer tissue accumulation with improved anticancer activity. The anticancer activity was found to be a result of the combination of PDT/PTT. In addition, the CD-treated groups did not show any histological changes in comparison with the control groups.^99^ In another study by Liu and colleagues, CD nanocomposites were prepared from soybean milk as a source of carbon *via* a facile, green, and one-pot approach. The developed fluorescent CDs showed efficient photothermal-mediated tumor ablation. The photothermal activity of the CDs was determined by irradiating the solution for 5 min with an 808 nm laser, and thermographs were captured. Compared with deionized water, the CDs showed an efficient photothermal effect. An increase in the photothermal activity with increasing CD concentration at an optimal distance of 1 to 3 cm was observed. Inspired by the above results, phantom photothermal imaging was performed in pork simulated tissue injected with CDs. A temperature increase of 25.3 °C was observed after irradiation for 10 min. PTT can effectively target malignant cells due to its specific effect.^201^ Meena and coworkers synthesized CQDs from medicinal plants such as *Azadiracta indica*,*Ocimum ienuiflorum*, and *Tridax procumbens* for tumor imaging and phototherapy. The photostability studies conducted on the prepared QDs revealed that they exhibit moderate to high photostability. Photothermal studies revealed an efficient temperature increase to 46 °C after irradiation with a 750 nm laser for 10 min.^202^

### 6.4. Nanotheranostic applications

BQDs are emerging as novel nanomaterials in the area of nanotheranostics, which merge diagnostic and therapeutic modalities into the same nanoplatform. The improved photobleaching, excellent photoluminescence and resistance to photobleaching exhibited by these platforms make them suitable for bioimaging applications. QDs allow efficient bioimaging, which allows their use in the early detection of cancerous cells by producing high-resolution images. In addition, they can be used for the specific detection of biomolecules related to cancer by functionalizing the surface. From a therapeutic perspective, biomimetic QDs facilitate the precise delivery of chemotherapeutics to the tumor site. The conjugation of QDs with chemotherapeutics and targeting agents allows for efficient chemotherapy with minimal side effects. BQDs can be efficiently utilized in PTT and PDT. In PTT, these nanomaterials can efficiently absorb NIR lasers and convert them to heat, which results in tumor ablation. The ability of QDs to produce ROS upon light activation leads to a cytotoxic effect on tumor tissues, making them suitable for PDT application.^203,204^

Among various carbon sources, natural biomass has proven to be an excellent candidate for preparing CD phototheranostic agents. In a study by Wen *et al.*, pheophytin, a magnesium-free chlorophyll derivative and a natural, low-toxicity product, was utilized as the raw carbon source for the microwave-assisted synthesis of CDs. The obtained CDs displayed an emission peak at approximately 680 nm with efficient generation of singlet oxygen and a quantum yield of 0.62. The ESR technique was employed for the determination of the ROS signal, and the singlet oxygen signal significantly improved upon irradiation with a 671 nm laser. The absorption spectrum of the CDs indicated that they have NIR light-sensitive activity with strong absorption in this region. The prepared CDs exhibited good photostability and maximum emission at approximately 680 nm, indicating their capability for NIR imaging. A cellular uptake study revealed strong fluorescence with laser exposure in 4T1 cells after incubation for 6 h. An MTT assay demonstrated efficient 4T1 cell killing upon irradiation with a 671 nm laser of 0.1 W cm^−2^ for 10 min. These results suggest the efficiency of the CDs for simultaneous imaging and PDT of tumors. *In vivo* evaluations of 4T1 tumor-bearing mice demonstrated complete tumor inhibition with efficient tumor accumulation and singlet oxygen production upon irradiation with a 671 nm laser.^205^

Thakur *et al.* developed theranostic GQDs from cow milk *via* a facile green chemistry approach. *In vitro* cytotoxicity evaluation revealed increased toxicity in MDA-MB-231 cells. The GQDs were biocompatible in nature at relatively high concentrations, indicating their suitability for cellular bioimaging applications. The GQDs were efficient both as pH-responsive drug carriers and as bioimaging platforms. Cellular localization studies have indicated the efficiency of milk-derived GQDs as imaging nanoprobes at lower concentrations.^108^ CDs developed from *Hypocrella bambusae* have been applied in bimodal (fluorescence and photoacoustic) image-guided PDT/PTT of cancer. The developed CDs exhibited efficient singlet oxygen generation and improved hyperthermia for combination PDT/PTT anticancer activity in comparison with the PDT-treated group alone. The quantum yield of the prepared CDs was 0.38, with a temperature increase of 26.9 °C at a concentration of 200 μg ml^−1^ upon irradiation with a 0.8 W cm^−2^ 635 nm NIR laser. Moreover, *in vivo* studies have demonstrated efficient anticancer activity with combined PDT/PTT.^99^

### 6.5. Photodynamic therapy

Photodynamic therapy (PDT) is a novel approach for cancer therapy that uses light-sensitive photosensitizers for efficient tumor ablation. The characteristic optical properties and desired biocompatibility of BQDs make them suitable candidates for PDT. They absorb lasers of different wavelengths and generate ROS, which are essential for photodynamic activity. BQDs generate singlet oxygen (^1^O_2_) through a photosensitization mechanism where upon excitation by light irradiation, the QDs transfer energy to ground-state molecular oxygen, converting it to the reactive singlet state. Studies have shown that N, S-doped or peptide-functionalized graphene quantum dots can reach singlet oxygen quantum yields as high as 0.95, making them highly effective for PDT, whereas nondoped or improperly processed dots might fail to generate significant amounts of ROS under similar conditions.^206^ Thus, both the intrinsic properties of BQDs and the extrinsic conditions during irradiation jointly determine their effectiveness as singlet oxygen generators in photodynamic therapy. The use of QDs for PDT offers different advantages over conventional photosensitive agents. Primarily, QDs demonstrate size-tuneable photoluminescence, allowing accurate control of light emission and absorption. This tunability allows for the selection of ideal wavelengths that can penetrate deeper into tissues, hence increasing the efficiency of PDT in solid tumors. Additionally, QDs have higher photostability than organic dyes do, making them trustworthy for longer exposure in therapeutic applications. The ability of BQDs to exhibit both photothermal and photodynamic activities further improves their therapeutic activity by combining ROS and heat generation with synergistic activity.^196^

Wen *et al.* prepared phenophytin-derived CDs for bioimaging and PDT of breast cancer. The ESR approach was used for monitoring ROS, and TEMP was employed as a ^1^O_2_ trap. The study revealed a significant increase in the ^1^O_2_ intensity upon irradiation with a 675 nm laser. In addition, the quantum yield was measured with DPBF, where a reduction in the absorbance intensity with prolonged exposure to radiation was observed. The quantum yield of the prepared CDs was 0.62. 4T1 cells were incubated with CDs to measure the fluorescence intensity, which represents their cellular uptake. Intense red fluorescence was observed in the cytoplasm of the CDs after incubation for 6 h, indicating efficient internalization. The ^1^O_2_ production capability of the prepared CDs at the cellular level was determined with DCFH-DA. Prolonging the irradiation time caused an increase in the green fluorescence signal, which indicates the successful generation of ^1^O_2_ at the cellular level. Moreover, *in vivo* studies have shown successful tumor ablation as a result of ^1^O_2_ generation upon laser irradiation.^205^

While the current literature focuses mostly on the impact of BQDs in breast cancer, increasing evidence shows their importance across all cancer types. By combining findings from other cancer studies, a better understanding of the use of QDs in the PDT of cancer can be obtained. Xue *et al.* prepared CDs from lychee exocarps and used them for the bioimaging and PDT of cancer. A significant production of ^1^O_2_ was observed upon irradiation with a 650 nm laser using DPBF as a sensing probe. *In vitro* studies on Bel7404 and HL7702 cells indicated the potential of the prepared CDs to be used as a PDT agent.^207^ In another study, Nasrin *et al.* prepared novel types of CDs from curcumin and folic acid *via* a hydrothermal process for the PDT of oral cancer. The prepared CDs significantly produced ROS upon two-photon NIR light activation. In addition, CD-treated DCFH-DA-stained H413 cells presented increased levels of ROS according to flow cytometry analysis.^208^

Recent advancements in the use of BQDs for breast cancer theranostics, including their sources, therapeutic agents, applications, and biological models, are summarized in Table 2.

**Table 2:** Recent studies on bioinspired quantum dots (BQDs) for breast cancer theranostics

QD	Source	Drug	Theranostic application	Cells/animals	Significance	Ref.
Ag_2_Te	Biomineralization	—	•CT imaging	4T1 murine breast tumor-bearing Kunming mouse	Efficient CT imaging agent for imaging tracking and monitoring during photonic hyperthermia	209
			•Tumour diagnosis			
			•Hyperthermia			
Au	Macrophage membrane	DOX	•Targeted chemotherapy	4T1 breast cancer	Efficient tracking of inflammation location. Multimodal therapy	210
			•Fluorescence imaging			
CQDs	*Hypocrella bambusae*	-	•Fluorescence/photoacoustic image-guided PDT	4T1, HeLa cell lines, 4T1 tumor-bearing female nude mice	Novel therapeutic platform for the combined chemo-photothermal therapy	211
			•PTT			
MoO_*x*_S_2−*x*_	Biomineralization	Camptothecin	•Chemotherapy	4T1, HeLa	Efficient therapeutic outcome for excellent therapeutic activities for image-mediated chemotherapy	212
			•Radical generation			
			•GSH deactivation			
			•H_2_S production			
			•Stimulus-responsive drug release			
FeS	BSA	—	•Image-guided phototherapy	4T1 tumor-bearing mice	Excellent tumour-ablation property	213
GQDs	Cow's milk	Berberine hydrochloride	•Multiexcitation imaging	MDA-MB-231, HeLa, L929	Multiexcitation mediated cellular imaging	108
CDs	Linseed	—	•Biosensing	MCF-7 cell lines	Excellent PL properties, thus displayed immense potential for cellular imaging	189
			•Bioimaging			
CDs	Eutrophic algal blooms	—	•*In vivo*/*in vitro* imaging	MCF-7 cell lines	Low cytotoxicity and excellent cell permeability	190
					Scalable method for the commercial production	

## 7. Biosafety concerns

The biosafety of QDs in cancer therapy and bioimaging is a valid concern. In the last two decades, many studies have investigated the influence of BQDs on animals and cells, with hundreds of studies published. Various experimental designs and QDs have been used in biocompatibility studies to assess their cytotoxicity. However, there is no complete model for describing the possible hazardous consequences of QDs. When the ligand shell of QDs is removed from the biological microenvironment, heavy metal ions are liberated into the cytoplasm, resulting in cytotoxicity.^214,215^ BQDs offer substantial advantages over traditional QDs in terms of long-term toxicity and environmental safety. Unlike chemically synthesized QDs, especially those produced in organic phases and containing toxic heavy metals, BQDs are prepared under mild, aqueous, and biologically controlled conditions, resulting in highly biocompatible and nontoxic nanostructures. The biological synthesis process avoids hazardous solvents and promotes the formation of pure, stable, and environmentally safe QDs without requiring postsynthetic surface modification for biocompatibility.^29^

The toxicity of QDs is influenced by their cellular uptake characteristics, so reducing their toxicity is essential for their use in biomedical applications. The biocompatibility of biomass-derived BQDs is primarily determined by their surface characteristics. A high concentration of polar functional groups such as hydroxyl, carboxyl, amine, and carbonyl groups originating from natural sources enhances water solubility, minimizes particle aggregation, and promotes efficient cellular uptake. Combined with the absence of toxic heavy metals, these attributes result in low cytotoxicity and minimal phototoxicity, as confirmed in cell models. Notably, BQDs doped with sulfur and nitrogen exhibit outstanding performance in bioimaging and drug delivery applications, offering superior photostability and exceptionally low toxicity.^32,110^

While inorganic QDs exhibit high fluorescence quantum yields and narrow emission spectra, they raise significant concerns regarding their toxicity and environmental persistence. In contrast, BQDs synthesized from carbonaceous biomass precursors offer environmentally friendly, water-soluble, and nontoxic alternatives with stable, tunable fluorescence and enhanced safety profiles. Their degradation in biological environments is facilitated by their organic structure and the presence of natural functional groups, making them suitable for applications that demand biosafety and sustainability.^216^ Most QDs are taken up nonspecifically by the reticuloendothelial system.^217^ Current research indicates that both size and surface modifications significantly influence the biodistribution and pharmacokinetic behavior of BQDs. For example, BQDs with a size less than 5 nm are quickly eliminated from the body due to renal filtration, with the size threshold for avoiding renal clearance being in the 5–6 nm size range.^218^ Furthermore, surface modifications can alter the distribution patterns of BQDs. Gold BQDs functionalized with macrophage-based macrovesicles were localized in the lungs of a model of breast cancer lung metastasis. In another study, red blood cell membrane-coated and cetuximab-loaded graphene QDs demonstrated improved penetration into tumors, achieving significant localization in the brain tumor region.^219,220^ Further research is needed to fully explore the mechanisms underlying their clearance from the body.

In biological environments, BQDs, particularly CQDs, can undergo photoluminescence quenching *via* multiple mechanisms, including static and dynamic quenching, photoinduced electron transfer (PET), fluorescence resonance energy transfer (FRET), and the inner filter effect (IFE). These processes are often triggered by interactions with biological ions (*e.g.*, Fe^3+^), proteins, or pH-sensitive species. Static quenching results from the formation of nonfluorescent ground-state complexes, whereas dynamic quenching involves energy transfer during the excited state.^221^ The safety of QDs is critical for their successful implementation in biomedical fields. While only a small percentage of novel imaging agents, such as fluorescent nanoparticles, have proven safe during preclinical trials, some advancements stand out. For example, Cornell dots—QDs functionalized with radioactive iodine (^124^I) and cyclic arginine-glycine-aspartic acid peptides—were recognized by the U.S. FDA in 2011 for clinical trials because of their demonstrated safety. Reports from 2014 indicated that no toxic or adverse effects were observed during these trials, and superior uptake of Cornell dots was noted in the cancer region. Despite these promising results, ensuring the safety of BQDs remains essential for their clinical translation.^222,223^

## 8. Challenges of bio-inspired quantum dots in drug delivery

Even though BQDs have promising properties, they encounter multiple obstacles that slow their transition into clinical applications. Among these methods, ensuring reliable and consistent target specificity is one. Even if surface modification of BQDs with targeting ligands can improve selectivity, the inconsistency in receptor expression in heterogeneous breast cancer cells reduces the effectiveness of BQD-based drug delivery. Moreover, since the vasculature of tumor cells varies, passive targeting through enhanced permeability and retention does not work effectively.^19^ Current synthesis methods for BQDs, including pyrolysis, hydrothermal, microwave-assisted, and electrochemical techniques, face challenges in achieving uniform particle sizes, consistent surface chemistry, and batch-to-batch reproducibility. The variability in biomass composition, reaction parameters (temperature, time, pH), and lack of precise control over nucleation and growth lead to inconsistent optical properties and quantum yields among batches. Moreover, methods such as hydrothermal carbonization often result in heterogeneous size distributions, and reproducibility is difficult to maintain without standardized precursor treatment and synthesis conditions.^224^

Potential toxicity and immunogenicity are other concerns of BQDs. Compared with conventional QDs, bioinspired quantum dots are safer, but some formulations contain small amounts of heavy metals such as cadmium, which raises concerns about long-term toxicity risks.^19^ However, it has been suggested that target specificity can be increased by conjugating a receptor ligand to a nanoparticle.^225^ Further research is needed to investigate the immune activation and organ accumulation of the degradation products of BQDs in the body.^19^ Ensuring the stability of BQDs in physiological environments is another key concern. Although current studies suggest that many BQDs elicit a minimal immune response, comprehensive immunotoxicological evaluations are still lacking. Strategies such as PEGylation or biomolecule shielding can reduce immune recognition and improve circulation time. Therefore, while BQDs are promising for biomedical use, their immunogenicity must be systematically assessed for safe clinical translation. The bioavailability and therapeutic performance of BQDs are diminished because of their accumulation in body fluids. The minimal therapeutic effect can also be the result of the premature release of the drug from the BQDs, which is another challenge that remains to maintain controlled release. Scientists have been actively investigating this topic to improve structural integrity and prolong retention time in the bloodstream.^19^ The heterogeneity of biomass feedstocks significantly impacts the reproducibility of BQD properties. Biomass derived from different sources, such as agricultural wastes, wood types, or plant parts, varies in its chemical composition, structure, and nutrient content. These inherent differences introduce complexity to the quantum dot synthesis process, making it difficult to consistently regulate the size, structure, surface chemistry, and, consequently, the optical and electronic properties of the resulting BQDs. For example, variations in lignin, cellulose, hemicellulose, and nitrogen contents in feedstocks can lead to fluctuations in mass yield and quantum yield during synthesis.^21^

The clinical translation of BQDs is still in the budding stage. Mass production and reproducibility are the main hindrances to this.^226^ The lack of scalability of conventional eco-friendly synthesis methods can cause size variation in BQDs, variation in surface characteristics and fluorescence between batches. These variations complicate regulatory approval. Additionally, the time-consuming and costly process of prolonged preclinical and clinical validation hinders the clinical translation of BQD-based interventions.^20^ However, BQDs offer exciting advancements, such as targeted drug delivery, imaging and theranostic applications in breast cancer treatment, for clinical translation, overcoming key challenges is crucial. Future research should emphasize optimizing the synthesis methods, stability, minimizing toxicity, and side effects and making them available at a large scale for efficient clinical translation.

## 9. Clinical trials

In a clinical trial specifically targeting breast cancer conducted by Al-Azhar University by Abdellatif *et al.*, veldoreotide, a somatostatin analogue, was selected as a potential candidate that is effective in delivering chemotherapy medications intended for targeting and bioimaging cancerous cells because interactions with somatostatin receptors (SSTRs) do not lead to immunogenicity *in vivo*. The primary objective of this endeavour was to formulate CdS/ZnS core–shell structured QDs functionalised with carboxylic acid (QDs-COOH) as a topical cream that would eventually be implanted deeply in the breast periphery. These QDs were nanoparticles coated with VELD as SSTR agonists and were found to have efficient bioimaging and anticancer properties. A sulforhodamine B-based assessment was used to evaluate growth inhibition. The quantity of fluorescent QDs-VELD was determined on the basis of QD fluorescence in the periphery of the breast, as measured by flow cytometry. The visual identification of malignant breast cells was used to quantify growth inhibition. Women between the ages of 25 and 60 who have undergone a breast biopsy within 60 days and are diagnosed with metastatic breast carcinoma with estrogen receptor (ER) concentrations ≥ 10% that have been confirmed by histology are included in the study. In addition to meeting certain blood parameter requirements, participants must have received conventional regional radical therapy, regardless of whether they received neoadjuvant/adjuvant chemotherapy or radiation. People with comorbidities that impact sex hormone levels, patients who have received adjuvant therapy in the past or are now receiving it, and those with disorders that make long-term follow-up difficult are excluded. Furthermore, those who had serious liver or heart illness, recent thrombotic episodes, or any other cancer during the previous five years—aside from carcinoma of the basal cells or even cervical *in situ* carcinoma—were not included. Women of reproductive age who do not utilize the recommended form of contraception, as well as those who are pregnant or nursing, were also not eligible. The last known status of this clinical study was reported in 2019.^227^ The sustainability and biocompatibility of these quantum or corneal dots (QDs) are significant concerns, especially in regard to those that contain heavy metals such as cadmium. To ensure that QDs can be appropriately utilized in clinical settings, research is necessary to create safer substitutes devoid of cadmium and other hazardous substances. Further research is needed to verify the safety of these nanoparticles for use in biomedical applications because of their possible toxicity. The demand for QDs that do not degrade the well-being of patients over time emphasizes this necessity.^228–230^ Researchers are looking into conjugating QDs with different biomolecules to increase their targeting and therapeutic delivery, resulting in enhanced imaging and treatment tactics. Improved targeting and delivery mechanisms likewise remain essential.^231,232^ For QD-based technologies, the path from the laboratory to the clinic is paved with obstacles. Preclinical research, which is mainly performed in animal models, has shown encouraging outcomes, but applying these discoveries to cases involving humans is difficult. In addition to proving the safety and clinical effectiveness of QDs, this procedure involves the development of scalable production methods for their broad clinical application. To successfully incorporate QD-based technologies into routine cancer care procedures, thorough clinical trials and regulatory approval are essential phases in this course of action.^233,234^Table 3 summarizes the results of clinical trials involving bioinspired QDs.

**Table 3:** Clinical trials involving bioinspired quantum dots

Sr. no.	Clinical trial number	Study title	Disease	Treatment	Year	Phase	Sponsor	Ref.
1	NCT04138342	Topical fluorescent nanoparticles conjugated somatostatin analogue for suppression and bioimaging breast cancer	Breast and skin cancer	Drug: QDs coated with veldoreotide	2019–2022	Phase 1	Al-Azhar University	227, 235 and 236
2	NCT04390490	Clinical trials of photoelectrochemical immunosensor for early diagnosis of acute myocardial infarction	Acute myocardial infarction	Device: Si nanowire photoelectrochemical immunosensor in conjunction with graphene quantum dots (the photoelectrochemical immunosensor will be utilized for identifying the level of cardiac troponin I as test group)	2020–2023	N.A	Bin He	237
3	NCT05841862	Intravitreal quantum dots (QD) for advanced retinitis pigmentosa (RP) (QUANTUM)	Retinitis pigmentosa	Device: 2C-QD (single-dose intravitreal injection)	2023–2025	N.A	2C Tech Corp	238

## 10. Regulatory aspects

To ensure that QDs may be used safely in biomedical applications, their toxicity must be thoroughly assessed. Cornell University created the first quantum dots, also called Cornell dots (Cdots), which consist of fluorescent core shells composed of silica nanoparticles smaller than 8 nm in diameter and enclosed organic dyes covalently. The use of Cdots in the treatment of melanoma demonstrated the safety and efficacy of quantum dots. With respect to their potential applications or uses, QDs can be divided into three broad groups: class I QDs are used in electronics and computing; class II QDs are used as diagnostic or screening agents, medical devices, and imaging equipment; and class III QDs are associated with pharmaceuticals and nanomedicines.^239^ A few examples of marketed QDs include VIVODOTS®, which are commercially available QDs marketed in two forms, cadmium-based or cadmium-free, which are utilized to map tumorous tissue without removing healthy tissue during surgery. The metal–oxide–semiconductor QD HEATWAVETM was initially developed by the Nanoco Group platform. It can measure blood levels of glucose, bilirubin, oxygen, and haemoglobin. For noninvasive diagnosis, these QDs can also be adjusted to measure the concentration of particular elements in a patient's blood.^240^ NNCrystal US Corporation manufactures NN-Labs®, which are QDs that are effective in cellular and *in vivo* imaging as well as molecular detection.^241^ According to reports from 2014, enhanced reception of QDs at melanoma locations was noted during human clinical studies, and no harmful or negative consequences were reported. However, the safety of QDs is crucial for their clinical implementation.^242^ Only a small portion of novel imaging agents, such as fluorescent particles, have demonstrated safety in preclinical testing. Notably, cyclic arginine-glycine-aspartic acid peptide-functionalized QDs (Cornell dots) and radioactive iodine (^124^I)-labelled QDs have been shown to be safe for use and were authorized for human clinical trials by the US Food and Drug Administration (FDA) in 2011.^243^ To ensure their incorporation into standard breast cancer screening and treatment protocols, extensive clinical trials are needed to determine the safety, efficiency and regulatory authorization of bioinspired QD-based technologies. Another crucial area of focus is the implementation of QDs with personalized medicine, as QDs have the potential to enable personalized treatment plans through enhanced molecular profiling and biomarker detection, which might help develop customized and effective breast cancer interventions.^244^ Navigating these obstacles and promoting innovation in cancer treatment will require persistent coordination and contributions across researchers, regulatory bodies, and medical professionals. Regulations must continue to be periodically revised as research advances to account for the special characteristics of quantum dots and still comply with strict safety and effectiveness requirements.

## 11. Future perspectives and conclusions

The advancement of a technology-driven global environment has led to the development of numerous nanomaterials and practices that enhance quality of life and simplify daily activities. Among these innovations, nanotechnology stands out as a transformative force in science. This field involves the use of materials with dimensions in the nanometres range, enabling them to display exceptional properties and find applications across various disciplines. Nanotechnology, which is inherently multidisciplinary, has applications in virtually every domain. Within this realm, bioinspired QDs have garnered significant attention over the past decades. These nanoscale materials, which typically range in size from 1–10 nm, possess remarkable properties, including optical, electrical, biological, chemical, physical, and structural characteristics.

Bioinspired QDs have shown significant promise in the biomedical sector, particularly for diagnosing and treating diseases. Biogenically synthesized QDs are particularly effective in identifying small tumors and can penetrate deeper into cancerous tissues, increasing their efficacy in cancer detection and therapy. By modifying the surfaces of bioinspired QDs with antigens, antibodies, or proteins through biomimetic molecular processes, these nanomaterials can be customized for theranostic applications, allowing for the precise targeting of cancer cells. The availability of different functional groups on bioinspired QDs improves their solubility and dispersibility, thereby increasing their use in biomedical applications. In particular, bioinspired QDs have made notable advancements in the imaging, diagnosis, and therapy of breast cancer. They offer enhanced specificity, targeted therapeutic capabilities, and the potential for personalized medicine. Despite these advancements, challenges persist, such as concerns over biocompatibility, safety—particularly owing to cadmium content—and the need for robust clinical validation. Further research should prioritize the development of nontoxic, biocompatible QDs and rigorous clinical trials to establish their safety and efficacy. Compared with traditional QD synthesis, large-scale biomass harvesting and processing for BQD production typically offers more sustainable and environmentally friendly advantages, particularly because it makes use of organic or agricultural waste that would otherwise end up in landfills or worsen the environment. In addition to reducing the need for harmful byproducts and toxic reagents, biomass-based BQD synthesis promotes a circular economy by turning trash into useful nanomaterials that can also help with environmental remediation applications. Energy and water consumption as well as the production of residual waste during synthesis are examples of processing impacts, particularly when procedures are not optimized for resource efficiency or emission control.^245^

Advances in machine learning (ML) have proven highly effective in optimizing the synthesis of carbon-based nanomaterials, including QDs. ML algorithms can analyse experimental data to uncover relationships between synthesis parameters such as precursor type, reaction temperature, and time and the resulting optical and structural properties.^246^ Current biomass-based synthesis techniques, such as hydrothermal, microwave-assisted, and pyrolysis methods, offer promising scalability because of their low cost, simplicity, and eco-friendliness. However, challenges remain in maintaining batch-to-batch consistency, control over particle size, and surface functionality at larger scales. While energy consumption and byproduct management must be balanced at industrial scales, continued enhancements in process control and purification solutions reinforce the scalability of BQDs, making these methods well suited for commercial applications without compromising material quality.

Moreover, biogenically synthesized QDs demonstrate outstanding photothermal conversion efficiency and stability. They are also valuable for generating high-resolution T1-weighted MR images because of their superior longitudinal relaxation times. Consequently, bioinspired QDs are increasingly being employed in the creation of point-of-care and lab-on-a-chip sensors, facilitating rapid disease detection. While the potential of quantum dots in revolutionizing science is immense, comprehensive preclinical and clinical studies are essential to understand their behaviour within biological systems. This is a prerequisite for their approval in cancer diagnosis and treatment. This review aims to provide an in-depth understanding of the biomedical applications of biogenically synthesized QDs. It covers their unique properties, surface modification techniques, and diverse biomedical uses in breast cancer while also addressing their toxicological aspects, making this an invaluable resource in the field.

## Author contributions

Soji Soman: writing – original draft, writing – review & editing, conceptualization, methodology, data curation. Sanjay Kulkarni: writing – original draft, writing – review & editing, conceptualization, methodology, data curation. Farhath Sherin: writing – original draft, methodology, data curation. Amrita Arup Roy: writing – original draft, methodology, data curation. Anoushka Mukharya: writing – original draft, methodology, data curation. Rahul Pokale: writing – original draft, methodology, data curation. Srinivas Mutalik: investigation, conceptualization, project administration, resources, supervision, validation, writing – review & editing.

## Conflicts of interest

The authors declare no competing interests.

## Data Availability

This is a review paper and as such it does not include any primary datasets. All the data we discussed and analyzed within this review are derived from the published studies and literature references in the manuscript.
